# VarWalker: Personalized Mutation Network Analysis of Putative Cancer Genes from Next-Generation Sequencing Data

**DOI:** 10.1371/journal.pcbi.1003460

**Published:** 2014-02-06

**Authors:** Peilin Jia, Zhongming Zhao

**Affiliations:** 1 Department of Biomedical Informatics, Vanderbilt University School of Medicine, Nashville, Tennessee, United States of America; 2 Center for Quantitative Sciences, Vanderbilt University Medical Center, Nashville, Tennessee, United States of America; 3 Department of Cancer Biology, Vanderbilt University School of Medicine, Nashville, Tennessee, United States of America; 4 Department of Psychiatry, Vanderbilt University School of Medicine, Nashville, Tennessee, United States of America; Indiana University, United States of America

## Abstract

A major challenge in interpreting the large volume of mutation data identified by next-generation sequencing (NGS) is to distinguish driver mutations from neutral passenger mutations to facilitate the identification of targetable genes and new drugs. Current approaches are primarily based on mutation frequencies of single-genes, which lack the power to detect infrequently mutated driver genes and ignore functional interconnection and regulation among cancer genes. We propose a novel mutation network method, VarWalker, to prioritize driver genes in large scale cancer mutation data. VarWalker fits generalized additive models for each sample based on sample-specific mutation profiles and builds on the joint frequency of both mutation genes and their close interactors. These interactors are selected and optimized using the Random Walk with Restart algorithm in a protein-protein interaction network. We applied the method in >300 tumor genomes in two large-scale NGS benchmark datasets: 183 lung adenocarcinoma samples and 121 melanoma samples. In each cancer, we derived a consensus mutation subnetwork containing significantly enriched consensus cancer genes and cancer-related functional pathways. These cancer-specific mutation networks were then validated using independent datasets for each cancer. Importantly, VarWalker prioritizes well-known, infrequently mutated genes, which are shown to interact with highly recurrently mutated genes yet have been ignored by conventional single-gene-based approaches. Utilizing VarWalker, we demonstrated that network-assisted approaches can be effectively adapted to facilitate the detection of cancer driver genes in NGS data.

## Author Summary

A cancer genome typically harbors both driver mutations, which contribute to tumorigenesis, and passenger mutations, which tend to be neutral and occur randomly. Cancer genomes differ dramatically due to genetic and environmental factors. A major challenge in interpreting the large volume of mutation data identified in cancer genomes using next-generation sequencing (NGS) is to distinguish driver mutations from neutral passenger mutations. We propose a novel mutation network method, VarWalker, to prioritize driver genes in large scale cancer mutation data. Applying our approach in a large cohort of lung adenocarcinoma samples and melanoma samples, we derived a consensus mutation subnetwork for each cancer containing significantly enriched cancer genes and cancer-related functional pathways. Our results indicated that driver genes occur within a broad spectrum of frequency, interact with each other, and converge in several key pathways that play critical roles in tumorigenesis.

## Introduction

Next-generation sequencing (NGS) technologies have enabled genome-wide identification of somatic mutations in large scale cancer samples. One major challenge in interpreting the large volume of mutation data is to distinguish ‘driver’ mutations from numerous neutral ‘passenger’ mutations to facilitate the identification of targetable genes and new drugs. So far, the most widely adopted method is to search for highly frequently mutated genes within one cancer type [1], [2]. Although effective in many cases, frequency-based approaches suffer from disadvantages such as lack of power to detect infrequently mutated driver genes and failure to incorporate functional interconnections and regulations among genes. Recently, many new methods have been reported. For a more comprehensive review, please refer to [3], [4].

The complex features of mutations derived from NGS data present great challenges for computational approaches, both genetically and technically. First, the probability that a gene is mutated in a sample, i.e., the gene-based mutation rate, is influenced by both genetic and environmental factors. In this study, we only consider single nucleotide variants (SNVs) and small insertions and deletions (indels), and we define a mutant gene (abbreviated as MutGene) if it harbors at least one non-silent deleterious SNV or indel (see Materials and Methods). Assuming that mutations occur randomly across the genome, long genes have a better chance of harboring mutations (e.g., the gene *TTN*). Other factors, including sequence context, GC content, replication timing, chromatin organization, and alterations in mutation repair systems [5], [6], [7], as well as personal lifestyle and mutagen exposure period and level, have an impact on the gene-based mutation rate in an individual. Second, mutation ‘hotspot’ families, among other factors, often contribute many genes to the list of top candidate genes that are ranked by frequency. For example, genes from the olfactory receptor family are frequently mutated in many cases [1], including both normal and disease samples [8]. However, it remains unknown whether these mutations, or only some of them, are disease-related. Finally, sequence errors exist; however, large scale validation is still a challenge in NGS projects that involve hundreds of cancer samples. Since all of these factors accumulate non-clinically related events in mutation data, these biases should be considered when developing new approaches to prioritizing driver mutations.

An alternative approach to detect possible driver genes overlays the mutation genes in the context of biological pathways or protein-protein interaction (PPI) networks and then performs integrative analyses to identify significantly altered pathways or subnetworks. In cancer, functional pathways or biological networks are frequently interrupted in many patients [9], and their gene components present mutually exclusive or co-occurring patterns [10]. To date, only a few studies have searched the cooperative mutation modules underlying cancer [11], [12]. Notably, the incorporation of other large scale genetic and/or genomic data, such as mRNA abundance [13] and methylation data [14], can greatly improve the detection of driver genes. However, these datasets are not always available for the same patient cohort in large-scale sequencing projects, creating both challenges and a high demand to develop comprehensive approaches that can prioritize driver genes from mutation data.

In this work, we propose VarWalker, a network-assisted approach that aims to prioritize potential driver genes and better interpret mutation data in NGS studies. Our goal is to develop a tool that can address the huge variations among cancer samples as well as implement conventional approaches in modern NGS data analysis. VarWalker performs sample-specific filtering and implements the Random Walk with Restart (RWR) algorithm to search for frequently interrupted interactions between MutGenes and their interactors. We argue that if an interaction is interrupted by mutations in one or two of its linking proteins across many samples, this interaction has a higher chance to be biologically important than an interaction in which only one protein is disrupted by mutations. We demonstrated VarWalker in two recent large-scale NGS benchmark studies: one involving 183 matched tumor/normal LUAD samples [15] and the other involving 121 matched melanoma samples [16]. In each cancer, we derived a consensus mutation network, which was shown to be significantly enriched with known cancer genes and cancer-related functional pathways. Importantly, we not only identified highly recurrently mutated genes, but also well-known yet infrequently mutated genes, thereby demonstrating the usefulness of VarWalker to prioritize driver genes from NGS data.

## Results

### An overview of the VarWalker approach

**Figure 1:**
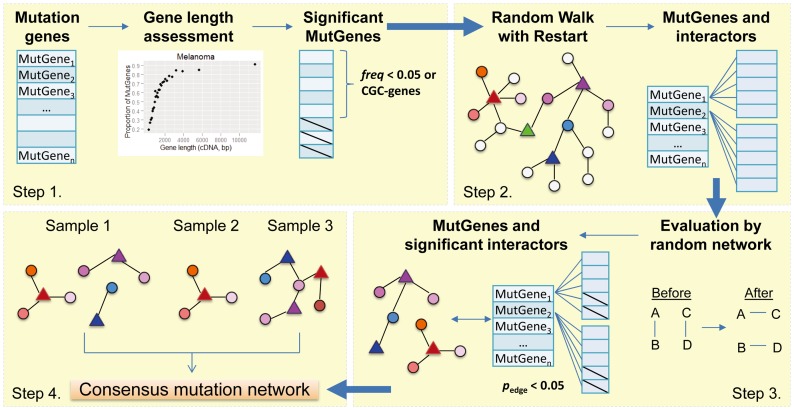
Flowchart of VarWalker. The pipeline has four steps, with steps 1–3 implemented in each sample and step 4 implemented in the whole cohort. In step 1, the mutation genes for each sample (MutGenes, defined as those with ≥1 deleterious somatic mutation in coding regions) are first assessed to compute a probability weight vector (PWV) by fitting a generalized additive model. A weighted resampling test based on the PWV is then performed to build a null distribution in which genes occur at random. Genes with *freq*≥0.05 are filtered, unless they are CGC genes, resulting in a set of significant MutGenes for each sample. In step 2, Random Walk with Restart (RWR) is initiated for each of the significant MutGenes, and their top interactors are collected. In step 3, these interactors are evaluated in 100 random networks generated with the same topological structures and performed using the same RWR algorithm. Interactors that are not observed by random chance, i.e., *p*_edge_<0.05, are then denoted as significant interactors and retained. In step 4, all significant interactors and interactions from each sample are pooled together, and a consensus mutation network is constructed.

The detailed description of the VarWalker algorithm is provided in Materials and Methods. It has four steps (Figure 1). The first three steps are implemented within each single sample, and the last step is across multiple samples. In step 1, for each sample, VarWalker assesses the mutation probabilities of all human genes by fitting them to a generalized additive model based on the patient- (or sample-) specific mutational profile. A weighted resample-based test is then performed to filter passenger genes that occur largely due to random events across the genome. Genes occurring with a frequency of ≥5% in random datasets were suggested for filtration. Step 2 includes the execution of the RWR algorithm in each sample to search for the interactions among the filtered MutGenes in the human interactome. RWR has been proven to be sensitive in identifying disease candidate genes and has been successfully applied in disease-phenotype analyses [17], [18]. Here, the introduction of RWR in mutation data analysis reinforces the recognition that driver MutGenes tend to converge in functional pathways and interrupt the same biological processes frequently, while passenger MutGenes are more likely to occur randomly in the genome (as do their interactors in the whole interactome). This recognition enables us to consult both MutGenes and their close interactors and prioritize MutGenes based on their joint frequency. In step 3, considering the complex topological features of human interactome data, we introduce a randomization-based test to evaluate the candidate interactors utilizing 100 topologically matched random networks. Candidate interactors that remain significant (i.e., *p*_edge_<0.05) are collected to form a universal candidate pool. This step is also implemented in each sample, respectively. Finally, a consensus mutation subnetwork is constructed (step 4) by collapsing all sample-specific results.

Using the overall implementation principles described above, we rigorously examined several factors that may influence the results as well as several parameter tunings that can potentially improve the performance. Text S1 in the Supporting Information provides a detailed description of these evaluations. We implemented our method in the network data from the Human Protein Reference Database (HPRD), which serves as a good balance between completeness and biological inference.

### Cancer Gene Census genes have small shortest path distance in HPRD

The Cancer Gene Census (CGC) [19] is a continuous effort to collect cancer genes with mutations that have been causally implicated in cancer. CGC genes are widely used in many cancer studies for benchmark evaluation. We first explored the topological features of CGC genes in HPRD. In our downloaded version (03/15/2012), a total of 487 CGC genes are included, and 369 of them have protein interactions in HPRD. The examination of the distance (measured by the shortest path) among CGC genes and others showed that CGC genes tend to be located more closely to each other than other genes. Specifically, 263 out of the 369 (71.27%) CGC genes are directly connected, 96 (26.02%) have a shortest path of 2 from other CGC genes, and only 10 (2.71%) have a shortest path ≥3 from other CGC genes. In contrast, in the whole HPRD network, 2931 (33.43%) genes (including 263 CGC genes) directly interact with CGC genes, 4657 (53.11%) genes have a shortest path of 2 from CGC genes, 1038 (11.84%) have a shortest path of 3, and the remaining 142 (1.62%) genes have a shortest path >3 from CGC genes. In summary, 97.29% CGC genes are located within two steps from other CGC genes, whereas 86.54% of all human genes are located within this distance. Based on this observation, we conclude that known cancer genes such as CGC genes show a strong tendency to be more closely connected, which is consistent with previous observations that proteins involved in the same disease have an increased tendency to interact with each other [20]. Therefore, we implemented a filtering step to remove genes that are located far away from CGC genes (e.g., those with a shortest path ≥3).

We explored the number of MutGenes that are retained after each step. The largest proportion of MutGenes was removed during mapping of MutGenes onto the HPRD network. This removal resulted from a limitation of the current human PPI data knowledge. Specifically, during removal of genes located two steps away from CGC genes, an average of 88.06% (range: 66.67–100%) were kept in LUAD compared to the previous step. Similarly in melanoma, an average of 86.86% (range: 72.22–100%) were retained compared to the previous step. These results indicate that gene filtration based on distance from CGC genes does not filter a significant proportion of the MutGenes (Figure S2).

### Long genes are more frequently mutated in cancer

We first explored long genes in the two working datasets: a LUAD patient cohort using mutation data from whole-genome sequencing (WGS) and whole-exome sequencing (WES) [15] and a melanoma patient cohort using WES data [16]. The LUAD dataset contains 183 samples, among which 182 had at least one non-silent deleterious mutation. This dataset involves a total of 11,306 MutGenes. A detailed mutational profile can be found in Figures S3 and S4. We manually examined the MutGenes in these samples and observed the frequency-based approach has a strong preference towards long genes. As shown in Figure S5, of the 10 most frequently mutated genes in the LUAD samples, with the exception of *TP53* and *KRAS*, the remaining eight genes are relatively long when compared to the distribution of all human CCDS gene lengths. In contrast, we examined the least frequently mutated genes, i.e., those mutated in one LUAD sample, and surprisingly pinpointed several important cancer genes, including *MDM2*, *RAC1*, *AKT1*, and *CDK4*. These observations suggest cancer genes could mutate in a broad range of frequency spectrums, making it difficult for the frequency-based filtering approach to be effective.

**Figure 2:**
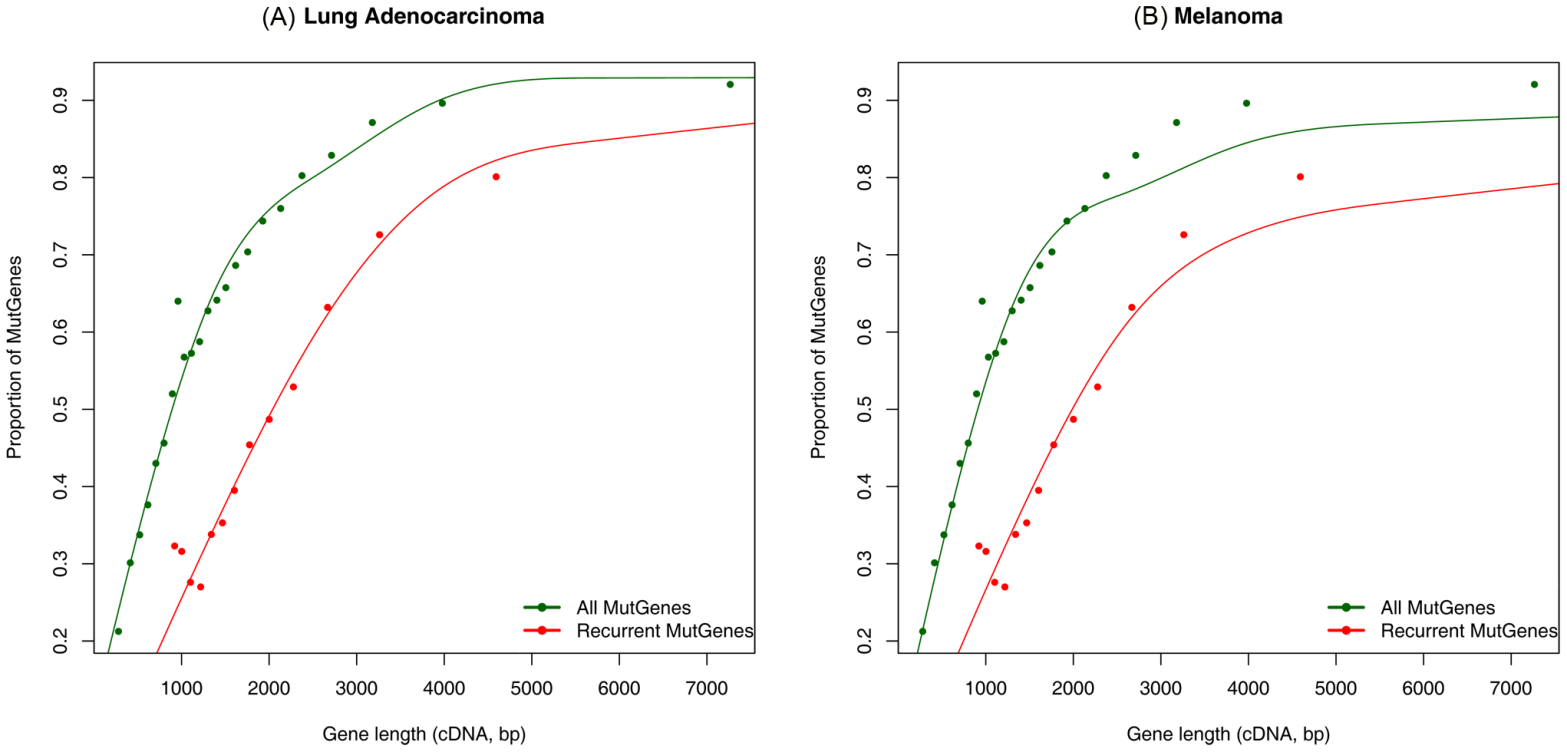
Distribution of mutation genes (MutGenes) as a function of gene length (cDNA length). (A) The proportion of MutGenes in lung adenocarcinoma (LUAD) samples versus gene length (cDNA length). The green line indicates all MutGenes in the 182 LUAD samples, and the red line indicates recurrent MutGenes, which occurred in ≥2 LUAD samples. (B) The proportion of MutGenes in melanoma samples versus gene length (cDNA length). The green line indicates all MutGenes in the 121 melanoma samples, and the red line indicates recurrent MutGenes, which occurred in ≥2 melanoma samples.

We then systematically examined the 11,306 MutGenes in the 182 LUAD samples. Among these MutGenes, 6878 were mutated in at least two samples (i.e., “recurrent MutGenes”) regardless of the mutation sites in these genes. Here, recurrent MutGenes differ from recurrent mutations, where the latter are defined as mutations that occur more than once at the same site. We hypothesize that genes that were mutated in only one sample are more likely to have their mutations attributable to random events. We then built two sets of MutGenes. Set one included all 11,306 MutGenes, and set two included all the recurrent MutGenes. We examined the gene length effects in these two MutGene sets by plotting the proportion of MutGenes versus their cDNA length. As shown in Figure 2, both sets have positive correlations with the cDNA length, but the trend was relatively weaker in set two. This analysis revealed that (i) the probability of observing MutGenes is indeed positively correlated with cDNA length, with longer genes more likely to be MutGenes; and, (ii) the correlation is reduced in recurrent MutGenes, yet is nontrivial (Figure 2A), indicating that even in recurrent MutGenes, random mutations still exist.

The same pattern was observed in melanoma samples (Figure 2B). A total of 121 melanoma patients had at least one non-silent deleterious mutation, involving 11,030 MutGenes that have CCDS IDs, 6852 of which were recurrent MutGenes. As shown in Figure 2B, both sets of MutGenes were positively correlated with cDNA length, and the recurrent MutGenes were less correlated, further supporting the necessity to perform gene length-based filtering.

### Application of VarWalker in LUAD samples

#### Build the consensus mutation network in LUAD

**Figure 3:**
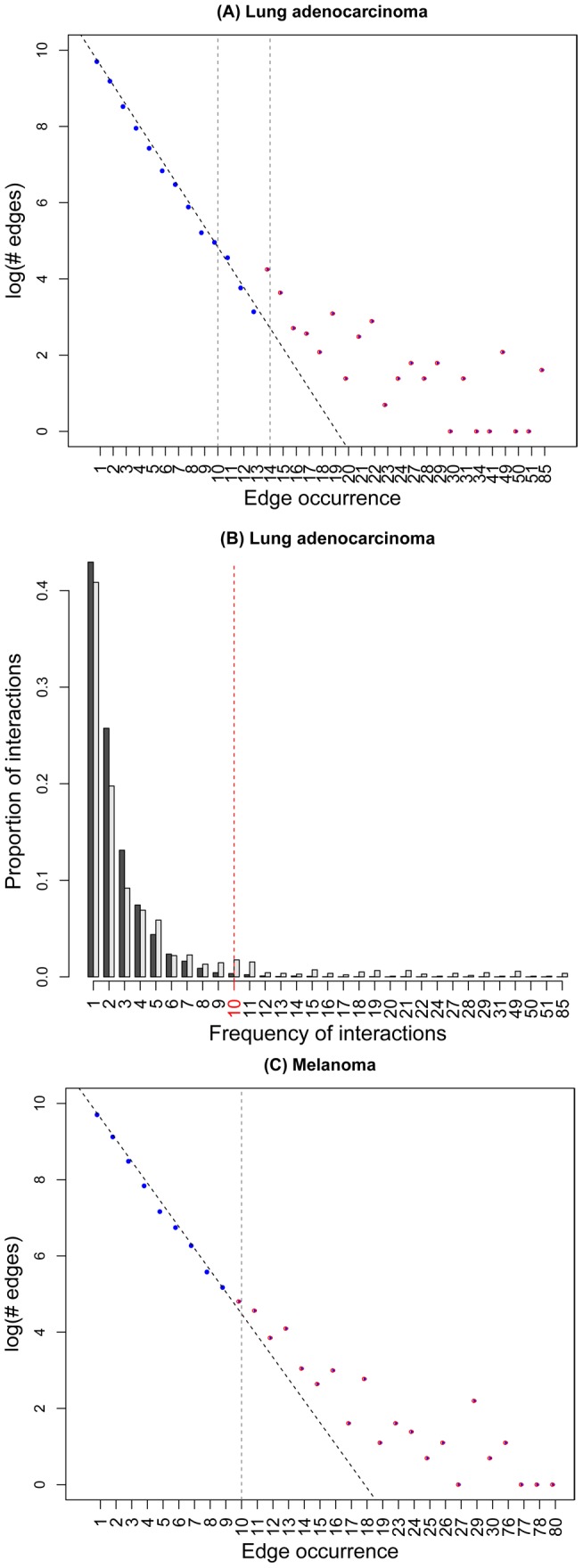
Distribution of significant interaction frequency. (A) Distribution of the number of edges (in a logarithmic scale) versus their occurrence in LUAD. The vertical line at 14 indicates the threshold at which the edges drifted away from the linear distribution. The vertical line at 10 indicates the threshold used for edge selection after a manual adjustment based on known LUAD genes. (B) The x-axis shows the frequency of interactions that were identified in 182 LUAD samples. The y-axis shows the proportion of interactions with the corresponding frequency in the x-axis. The black bar indicates the frequency for all significant interactions in all samples, while the grey bar indicates the frequency for the significant interactions involving any of the 52 known LUAD in HPRD. (C) Distribution of the number of edges (in a logarithmic scale) versus their occurrence in melanoma. The vertical line at 10 indicates the threshold at which edges drift away from the linear distribution; this threshold is used as the cutoff to select edges in melanoma.

For each of the 182 LUAD samples that had non-silent deleterious mutations, we applied VarWalker to all MutGenes and obtained a pool of significant interactions linking MutGenes and their significant interactors. To further condense this network, we selected highly recurrent interactions and built a subnetwork that is frequently mutated, which we denoted as a consensus mutation network. Notably, there are numerous methods to search for subnetworks. In our work, to avoid ambiguity in defining subnetworks [21], we focused on interactions that are frequently interrupted in many samples. We tabulated all edges according to their occurrence. As shown in Figure 3A, a linear correlation was observed between the number of edges (in a logarithmic scale) and their occurrence. We therefore fitted a linear regression model to the number of edges (in logarithmic scale) at each occurrence (R^2^ = 0.9978). The occurrence of edges in ≥14 samples drifted away from the linear distribution, and these edges were accordingly suggested for the construction of the consensus mutation network. However, in the case of LUAD, we had 57 known LUAD genes to facilitate the cutoff selection. As shown in Figure 3B, we manually adjusted the cutoff and chose 10 as the threshold in order to include more known LUAD genes. Interactions that occurred in ≥10 samples were collected to build the consensus mutation network for LUAD. This approach resulted in a subnetwork that included 307 interactions and 367 proteins encoded by MutGenes.

#### Validation of the LUAD consensus mutation network in an independent dataset

To validate the LUAD consensus mutation network, we retrieved The Cancer Genome Atlas (TCGA) LUAD somatic mutation data (denoted TCGA LUAD) and applied the same VarWalker procedure used for the discovery LUAD data. A total of 518 (as of 7/18/2013) TCGA LUAD samples were included (Table S1). After obtaining a significant interaction pool, the threshold for interaction selection was determined as 31 based on the interaction occurrence distribution (with no manual adjustment). Thus, interactions that occurred in ≥31 samples were collected to build the consensus mutation network, resulting in an evaluation mutation network consisting of 218 proteins and 197 interactions. Comparing the component genes in the discovery consensus mutation network with those in the evaluation mutation network, we found 116 genes (116/367 = 31.61% of the discovery network and 116/218 = 53.21% of the evaluation network) overlapped between the two networks. These overlapping genes were assessed as significantly higher than the expected level by a randomization test (*p*-value<1×10^−3^, Figure S6).

#### The LUAD consensus mutation network is enriched with known cancer genes

To evaluate these genes, we performed the following examination. (i) Comparison with known LUAD genes. As aforementioned, 31 out of the 52 known LUAD genes were included in our LUAD mutation network (5 known LUAD genes had no interaction annotation in the HPRD PPI network and were excluded). Note that the known LUAD genes had been used to adjust the threshold for interactions; therefore, the high proportion of known LUAD genes was expected. (ii) Comparison with CGC genes (independent test). A total of 369 CGC genes were present in the HPRD PPI network, among which 70 were included in our LUAD mutation network (*p*-value<2.2×10^−16^). (iii) Comparison with kinase genes (independent test), which are often cancer driver genes [22]. A set of 21 proteins in the mutation network were found to be encoded by kinase genes. These first three annotation categories include a total of 76 genes (20.71%), indicating that many of the genes in the mutation network are potentially cancer related.

#### The consensus mutation network in LUAD is enriched with cancer-related pathways

Our functional analyses of the consensus mutation network using the tool DAVID (Database for Annotation, Visualization and Integrated Discovery) [23] revealed a significant enrichment of multiple cancer-related pathways annotated by either the Kyoto Encyclopedia of Genes and Genomes (KEGG [24], Table S2) or Gene Ontology (GO) [25] biological process domains (Table S3). Of the top 15 significant pathways (*p*_Bonferroni_<10^−6^), 12 are directly related to cancer. Although expected, this result further demonstrated the enrichment of cancer genes in our mutation network.

#### Genes in the consensus mutation network correspond to hallmarks in LUAD

**Figure 4:**
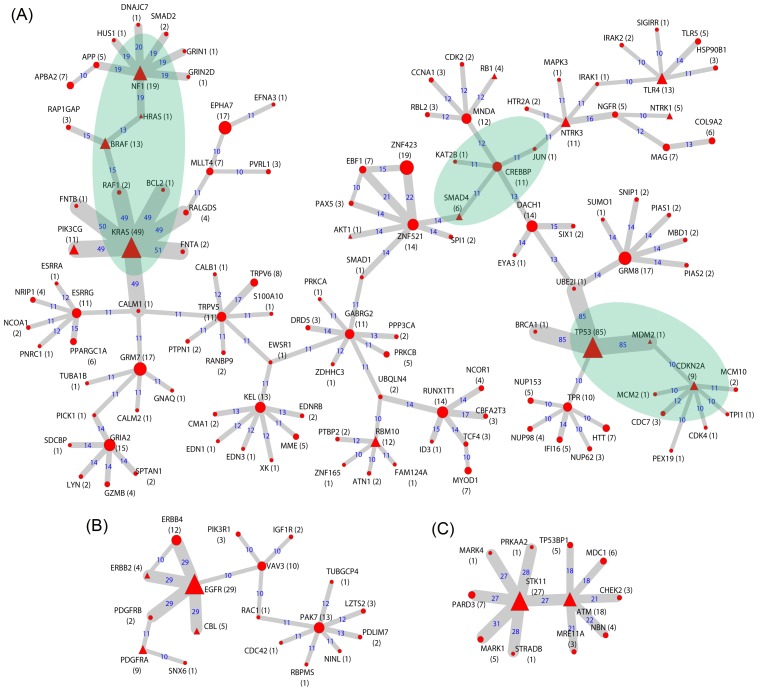
Selected subgraphs in the lung adenocarcinoma consensus mutation network. Node size is proportional to the number of samples harboring mutations in the corresponding gene (MutGene), as indicated in the parenthesis after the node name. The triangular nodes denote the proteins encoded by known LUAD genes (see Materials and Methods). Edge width is proportional to the number of samples in which the interaction is detected, which is also indicated by the number on each edge. Figures in (A), (B), and (C) show three selected subgraphs in LUAD.

Figure S7 shows the entire consensus mutation network, which consists of 67 subgraphs, including 30 non-orphan subgraphs (consisting of ≥2 edges) and 37 orphan subgraphs. We denote an orphan subgraph as one that consists of two nodes connected by one edge and are disconnected from any other subgraph(s) in the consensus network. Interestingly, nearly all the LUAD genes (29/31) were included in 9 of the 10 largest subgraphs, while only 2 known LUAD genes were included in 2 orphan subgraphs (Figure S7). Figure 4 shows the three largest subgraphs. For each subgraph, we performed a functional enrichment analysis of its genes. The results indicated that cancer-related pathways are primarily enriched in each of these subgraphs. Below, we briefly describe the subgraphs in decreasing order of the number of component genes.

The largest subgraph (Figure 4A) contains 126 genes, including 15 proteins encoded by known LUAD genes. Notably, these known LUAD genes could be highly frequently mutated (e.g., in more than 182×5% = 9.1 samples), such as *TP53* (in 85 samples), *KRAS* (49), *NF1* (19), *BRAF* (13), *TLR4* (13), *RBM10* (12), *PIK3CG* (11), and *NTRK3* (11). These genes could also be rarely frequently mutated (e.g., in <5% samples), such as *CDKN2A* (9), *SMAD4* (6), *NTRK1* (5), *RB1* (4), *AKT1* (1), *HRAS* (1), and *MDM2* (1). Functional enrichment analysis of this subgraph revealed a number of pathways related to cell signaling, receptor signaling, and cell cycle, among others (Table S4). Of special interest, three component interactions formed the central part of this subgraph, as highlighted in Figure 4A: (i) the proteins that mainly function in the EGF receptor signaling pathway (including HRAS, RAF1, BRAF, NF1, MAPK3, PRKCA, PRKCB, AKT1, PIK3CG, and KRAS, *p*_Bonferroni_ = 5.75×10^−3^); (ii) the proteins that function in the regulation of nuclear SMAD2/3 signaling pathways (SMAD2, SMAD4, MYOD1, CREBBP, JUN, SNIP1, NCOA1, NCOR1, CDK2, AKT1, CDK4, and KAT2B, *p*_Bonferroni_ = 1.17×10^−6^); and (iii) the proteins that play key roles in the p53 signaling pathway (MDM2, BCL2, RB1, TP53, CDK2, and CDK4, *p*_Bonferroni_ = 4.56×10^−5^).

The second subgraph (Figure 4B) consists of 18 nodes, including four known LUAD proteins, i.e., EGFR (mutated in 29 samples), PDGFRA (9), CBL (5), and ERBB2 (4). The focus of this subgraph is transmembrane receptors and receptor protein signaling pathways (Table S5), including the GO terms of transmembrane receptor protein tyrosine kinase activity (GO:0004714, *p*_Bonferroni_ = 3.45×10^−9^) and the related signaling pathway (GO:0007169, *p*_Bonferroni_ = 8.90×10^−11^), as well as various receptor binding processes (Table S5). Because many receptor proteins (e.g., EGFR, PDGFRA, PDGFRB, ERBB2, and ERBB4) are typically located in the upstream of cancer-related signaling pathways such as proliferation, cell death, and cell cycle progression [9], mutations in these genes are likely critical to cancer development.

The third subgraph (Figure 4C) includes 12 nodes spread around two hub proteins, STK11 (mutated in 27 samples) and ATM (mutated in 18 samples), both of which are encoded by known LUAD genes. Interestingly, the biological function of this subgraph is cell cycle and DNA repair (Table S6). Eight of the proteins in this subgraph participate in the cell cycle arrest process (GO:0007050, *p*_Bonferroni_ = 1.43×10^−8^), and six proteins function in the double-strand break repair process (GO:0006302, *p*_Bonferroni_ = 3.09×10^−8^).

#### Infrequently mutated genes were recruited in the mutation network

In addition to the highly frequently mutated genes (e.g., in ≥5% samples), there are several well-known cancer-related genes in the mutation network that are only mutated in a few samples. We specifically examined 204 (66.45%) interactions that linked an infrequently mutated gene (in <5% samples) and a highly frequently mutated gene (in ≥5% samples) and found 34/204 interactions that involved both interactors, each of which were encoded by known LUAD genes, CGC genes, or kinase genes (Table S7). These interactions were among 28 infrequently mutated genes (in <5% samples) and 16 highly frequently mutated genes (in ≥5% samples). Thus, these 28 genes are particularly promising, as they are cancer relavent genes and interact with highly frequently mutated genes, yet they would be ignored by a frequency-based approach. For example, the protein encoded by *BRCA1* (a CGC gene) interacts with TP53 (a high-frequency gene, known LUAD gene, and CGC gene), but it is only mutated in one sample. Similarly, the protein RAF1 (a CGC gene and kinase) interacts with both BRAF and KRAS (high-frequency genes), but it is only mutated in 2 samples. These genes provided a promising candidate list for driver genes in LUAD and warrant future investigation.

#### Comparision of consensus mutation networks for LUAD smokers versus never smokers

According to the smoking information of LUAD samples, there were 118 heavy smokers, 17 light smokers, 27 never smokers, and 21 with unknown status. To reveal potential differences between the consensus mutation networks of smokers and never-smokers, we applied VarWalker to the 135 smokers and 27 never smokers, respectively. The smokers' consensus mutation network contains 62 proteins connected by 47 interactions (Figure S8). For never smokers, the interactions have low frequency. We chose to manually select those interactions occurring in ≥3 samples, resulting in a never smoker-specific consensus mutation network with 15 proteins connected by 8 interactions. As shown in Figure S8, the two mutation networks are substantially different. In the smoker-specific consensus mutation network, the hubs include TP53 (mutated in 70 smoker samples), KRAS (42), STK11 (23), KEAP1 (20), EGFR (18), SNTG1 (16), ATM (15), and NF1 (15). In the never smoker-specific mutation network, we did not obtain many interactions, with *EGFR* being the most frequently mutated gene (mutated in 10 never smokers). Notably, *TP53* was mutated in 5 samples out of the 27 never smokers. However, its interactions did not have a high frequency; thus, it was not included in the consensus mutation network. Since the small sample size of never smokers is small, a more connected mutation network would be expected when large samples are available.

### Application of VarWalker in melanoma samples

The same procedure that was used in LUAD was applied to the 121 melanoma samples, all of which had MutGenes. Using the same criteria, we constructed a melanoma consensus mutation network, which contains 331 MutGenes involved in 301 interactions. We found that 65 of these 331 MutGenes are CGC genes, indicating a significant enrichment of cancer genes in the network (*p*-value<2.2×10^−16^, Fisher's Exact test). Further examination showed 15 kinase proteins in the network, most of which overlapped with CGC genes.

We also validated the melanoma consensus mutation network using somatic mutation data from the TCGA Skin Cutaneous Melanoma (SKCM) project. Many genes in the discovery consensus network were replicated (Table S1). In particular, 86 overlapping genes that account for 25.98% in the discovery dataset and 73.50% in the evaluation dataset were identified, which is significantly higher than expected by chance (*p*-value<1×10^−3^, Figure S6). Similar to the case of LUAD, these results demonstrated that cancer-related genes are effectively prioritized by VarWalker.

Functional enrichment analysis of the mutation network revealed many cancer-related signaling pathways (Table S8) and biological processes (Table S9), further indicating that the resultant network is enriched with cancer-related genes and regulation. For example, 12 of the 19 top significant KEGG pathways (*p*_Bonferroni_<10^−6^) are cancer-related (Table S8).

#### Consensus mutation network in melanoma

**Figure 5:**
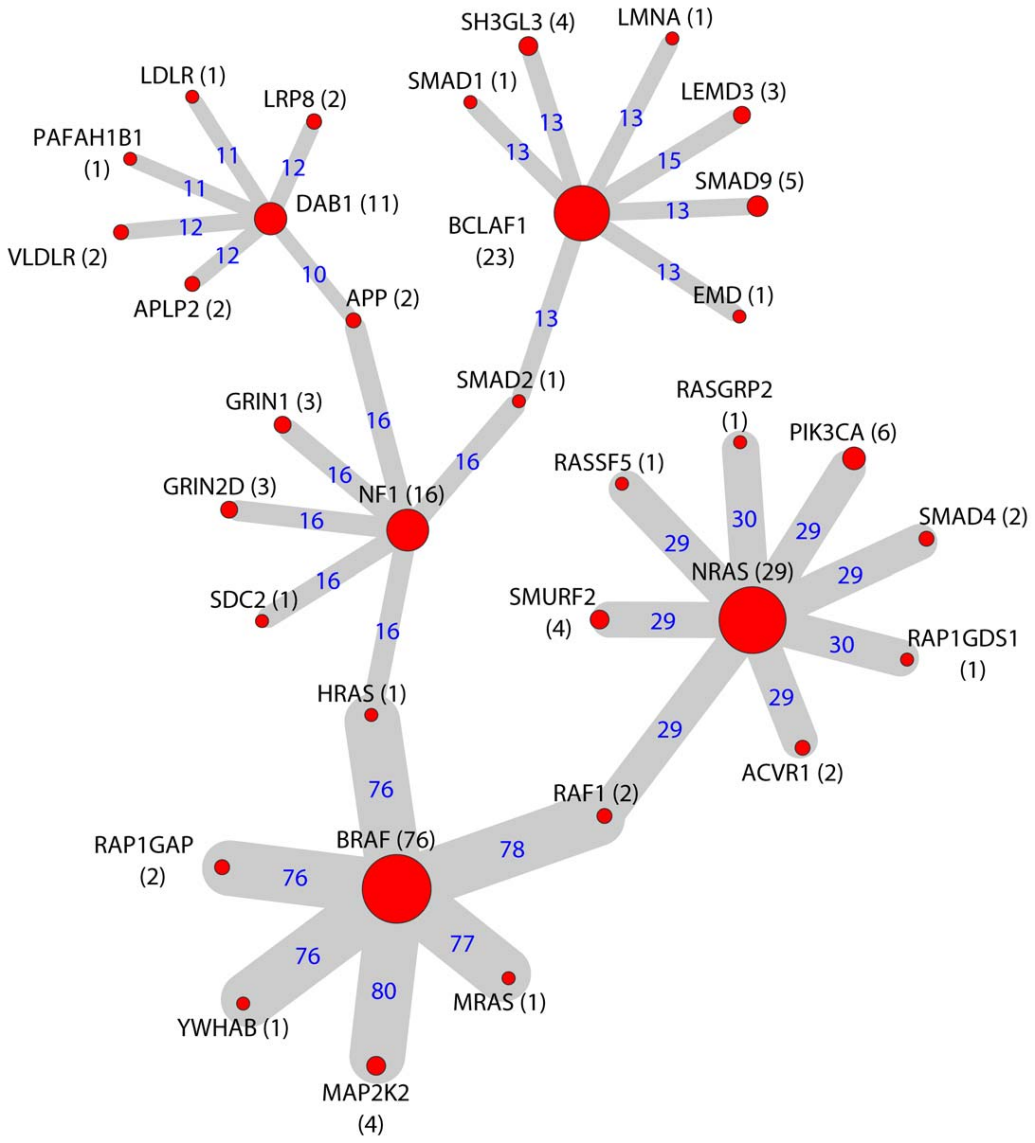
Selected subgraphs in the melanoma consensus mutation network. Node size is proportional to the number of samples harboring mutations in the corresponding gene (MutGene), as indicated in the parenthesis after the node name. Edge width is proportional to the number of samples harboring the interactions, which is also indicated by the number on each edge. For example, *NRAS* was mutated in 29 samples, *PIK3CA* was mutated in 6 samples, and the interaction between their protein products was found by RWR in 29 samples. In contrast, *RASGRP2* was mutated in one sample and the interaction between its protein product and NRAS was found by RWR in 30 samples.

As shown in Figures 5 and S9, the melanoma mutation network formed 50 subgraphs. Of them, 26 are non-orphan subgraphs and 24 are orphan subgraphs. We describe two subgraphs from the consensus network. The first subgraph, as shown in Figure 5, consists of 34 proteins spread across several hub nodes. These hub nodes have a substantially higher degree than the rest of the nodes in the subgraph, and among them are BRAF, NRAS, NF1, DAB1, and BCLAF1. *BRAF* and *NRAS* genes typically show a mutually exclusive mutation pattern in melanoma samples [12]. In our mutation network, proteins encoded by these two genes do not interact directly; rather, they connect through RAF1, a less frequently mutated gene (mutated only in 2 melanoma samples). In fact, these three proteins, BRAF, RAF1, and NRAS, play key roles in the Raf/MEK/ERK and PI3K/Akt cascades, which serve as the common upstream regulation of several important signaling pathways. Indeed, functional enrichment analysis of this subgraph revealed a number of significant pathways (Table S10) that involve the Raf/MEK/ERK and/or PI3K/Akt cascades, such as the Ras pathway (*p*_Bonferroni_ = 1.15×10^−4^), FGF signaling pathway (*p*_Bonferroni_ = 1.93×10^−3^), VEGF signaling pathway (*p*_Bonferroni_ = 1.56×10^−3^), and PDGF signaling pathway (*p*_Bonferroni_ = 5.26×10^−3^).

The second subgraph, which contains the largest number of component nodes (top of Figure S9), has its 95 nodes anchored to the known cancer proteins CTNNB1, APC, PTEN, TP63, MAPK4, MET, RAC1, and ROS1. A few proteins encoded by genes from the cadherin superfamily were linked to the subgraph through interactions with CTNNB1. The cadherin superfamily plays important roles in cell-cell adhesion and transfers information between two cells; its deregulation has been reported in tumorigenesis, cell migration, and invasion [26], [27]. Cadherin family gene changes have been observed at several levels, including germline and/or somatic mutations, dysregulated expression, and abnormal methylation levels [27]. In our network, CDH2, CDH6, CDH7, CDH9, CDH10, CDH12, CDH15, and CDH18 intensively interact with each other, and some of these proteins interact with the cancer proteins CTNNB1 and APC. The right section of this subgraph reflects the signaling events mediated by VEGFR1 and VEGFR2, including the protein KDR and its interactors (FLT1, FLT4, VEGFC, SHC1, MAPK3, MAPK1, PLCG1, PIK3R1, PRKACA, PTPN11, and PTPN6). Many of these genes are also part of the EGF/EGFR signaling pathway (*p*_Bonferroni_ = 1.38×10^−9^), FGF signaling pathway (*p*_Bonferroni_ = 7.45×10^−9^), and PDGFR-beta signaling pathway (*p*_Bonferroni_ = 1.40×10^−8^), indicating their correlated functional roles in signaling pathways. Finally, several proteins, starting from MET through CBL, SYK, VAV1, RAC1, and ending with PAK7 and its interactors, form part of the B cell receptor related signaling events (*p*_Bonferroni_ = 2.57×10^−15^). Note that RAC1 was reported as a novel mutation gene in the original work [16]. Here, we unveiled its mutation interaction context through our network analysis, which will likely provide deeper biological interpretation and generate new hypotheses for future studies.

### Comparison with single gene frequency based approach

We compared our results with those from the single-gene-based strategy. In our application of VarWalker in LUAD, we selected interactions that occurred in ≥10 samples. This approach resulted in 367 genes, 70 of which are CGC genes (70/367 = 19.07%). Using the single-gene-based strategy, we also selected genes that were mutated in ≥10 samples. This step resulted in 426 genes, 16 of which are CGC genes (16/426 = 3.76%), much less than those observed in the consensus mutation network. In melanoma, we also selected interactions that occurred in ≥10 samples, generating a consensus mutation network with 331 genes, 65 of which are CGC genes. Using the single-gene-based strategy, we obtained 404 mutated genes in ≥10 melanoma samples, 23 of which are CGC genes. The proportion of CGC genes obtained by the single-gene-based strategy (23/404 = 5.69%) is also smaller than the proportion obtained by VarWalker (65/331 = 19.64%). These comparisons clearly proved that our network-based approach is superior to the single gene frequency based strategy.

## Discussion

In cancer research, distinguishing between driver mutations, which contribute to tumorigenesis, and passenger mutations, which are mostly neutral and occur randomly, is extremely important to understand and design targeted therapies and treatments. We proposed an approach to prioritize candidate driver MutGenes and biological networks using individual or cohort NGS data. Our method VarWalker estimates the occurrence of mutation events in the genome according to approximated probabilities based on coding gene length. It implements gene-based filtering such that it can exclude genes that are mutated largely due to random events. VarWalker utilizes the Random Walk with Restart algorithm to search for interaction partners that are close to the mutation genes and assesses the resultant interactions using a comprehensive randomization test, thereby greatly reducing potential random interactors (e.g., those with high degrees). In summary, this method has the advantages of both filtering random mutation genes and detecting possible driver genes along with their functional interactions. Hence, it is promising for driver gene prioritization in the era of personalized medicine.

The applications of our method to both LUAD samples and melanoma samples revealed a mutation network for each of them. These mutation networks include a large proportion of known cancer genes and show the interconnections among the protein products of mutant genes. Interestingly, in each of the subgraphs within the consensus mutation network, we observed key components involved in cancer-related signaling pathways and biological processes. For example, in the LUAD mutation network, the three largest subgraphs focused on (i) the EGF receptor signaling pathway, the regulation of nuclear SMAD2/3 signaling pathways, and the p53 signaling pathway; (ii) transmembrane receptors and receptor protein signaling pathways; and, (iii) the cell cycle and DNA repair systems, respectively. The subgraphs in the melanoma mutation network revealed featured pathways such as the Raf/MEK/ERK pathway and receptor signaling pathways (e.g., EGF/EGFR, FGF, PDGFR-beta signaling pathways). The diversity of the component mutation genes in the mutation networks confirms the multifactorial and multigenic mechanisms underlying cancer. These observations also demonstrated the advantages of network-based approaches over frequency-based approaches in prioritizing cancer genes and revealing their functional impacts.

Comparison of the consensus mutation networks of LUAD and melanoma revealed 94 overlapping genes, 33 of which are also CGC genes (Figure S10). We performed a functional enrichment test of these 94 genes (Table S11) and found that most of them are enriched in protein binding categories or cancer-related signaling pathways. The most highly enriched GO terms are involved in enzyme binding (*p*_Bonferroni_ = 2.16×10^−13^), receptor binding (*p*_Bonferroni_ = 3.03×10^−13^), phosphatase binding (*p*_Bonferroni_ = 5.85×10^−9^), and kinase binding (*p*_Bonferroni_ = 1.76×10^−6^). The most significant pathways include the pathway of “influence of Ras and Rho proteins on G1 to S transition” (*p*_Bonferroni_ = 1.26×10^−9^), “signaling events mediated by VEGFR1 and VEGFR2” (*p*_Bonferroni_ = 1.74×10^−8^), and “tumor suppressor Arf inhibits ribosomal biogenesis” (*p*_Bonferroni_ = 1.01×10^−7^). Collectively, these results suggested that the overlapping genes between LUAD and melanoma mainly function in cell signaling.

The advantages of our approach are threefold. First, in contrast to single-gene-based mutation frequency, our method is based on the joint frequency of two interacting proteins; thus, at the same threshold of frequency, our method can detect moderately or even rarely mutated genes that fail the threshold individually. Second, our interaction-based method helps to filter out many randomly occurring passenger genes, as these genes are expected to be randomly distributed in the network and the chance that their interactors are mutation genes is smaller. Third, our mutation network shows the interactions and context of mutation genes, providing an interpretation to facilitate biological functional analysis in the future, such as further investigation of the novel gene *RAC1* in melanoma.

The limitations of our work, which could be improved in future investigations, are reflected in several factors that may impact the results. First, the method is sensitive to the reference network, though it could be flexibly selected. Currently, PPI network resources are comprehensive, but most of them are collected from large scale experiments [28], [29], [30], [31]. Functional correlation networks are valuable when representing biological knowledge and correlations among genes but are generally limited to genes that have already been annotated. As shown in Figure S1, a condensed mutation network was generated from the functional correlation network. This consensus network recruited 22 known LUAD genes, fewer than the 31 known LUAD genes that were recruited in the HPRD-based mutation network. Future expansion of biological networks is expected to improve the detection of mutation networks.

Second, the threshold we used to select interactions, i.e., 10 for both LUAD and melanoma samples, is a trade-off between accuracy and recall rate. Decreasing this threshold value would recruit more cancer genes, but it would also introduce false-positives. Currently, we propose to fit a linear regression model between the number of edges and the edge recurrence. This strategy works in most cases, including the independent TCGA LUAD and SKCM datasets. In practical applications, expert guidance could help to further refine the selection of candidate genes, e.g., in the case of LUAD dataset [16]. In future work, we plan to optimize the selection of interactions by making it threshold-free.

Third, we may improve the mutation recurrence (*mr*) index through the use of more sophisticated statistical tests and by including protein domain information (details of the *mr* index is described in Methods and Materials). In our work, we examined the resultant mutation networks either with or without applying the criterion of *mr*<1.05 in LUAD samples. In the latter case, the recall rate of LUAD genes increased by 4%; however, this application also led to 20% more proteins recruited in the final mutation network and, correspondingly, greatly decreased the specificity. Taken together, the parameter *mr* performs satisfactorily in our work.

In summary, we present a sample-specific mutation network analysis method to prioritize cancer driver genes using the mutation profiles generated in NGS projects. Our method will be useful for investigators who explore cancer genes through rapidly emerging NGS applications in cancer research and personalized medicine. It can also be applied to explore functional mutations in other complex diseases or traits. The source code in R is available at http://bioinfo.mc.vanderbilt.edu/VarWalker.html.

## Materials and Methods

### Datasets

#### Lung adenocarcinoma mutation data

The lung adenocarcinoma mutation dataset is from a recent NGS study of 183 LUAD samples and their matched normal tissues [15]. Among them, 159 were sequenced by WES only, 1 by WGS only, and 23 by both WES and WGS. The called mutations from the 23 samples using both platforms were employed for cross-platform validation, and the validation rate was shown to be high (97–98% for substitutions and 84–86% for indels) [15]. Therefore, although the mutation data has not been completely validated through traditional Sanger resequencing, the quality of the data was estimated to be high. The samples include several levels of smokers ranging from never-smokers to heavy smokers. Thus, the mutational profile for each patient varies dramatically. More details can be found in the Supporting Information, Text S1.

The authors of the original work [15] nominated 25 significantly mutated genes using the software InVEx. Furthermore, they collected 19 well-known LUAD genes based on an expert review of the previous studies, 6 genes based on related copy number variation data, and 22 genes from two previous large-scale sequencing studies of LUAD [9], [32]. We manually extracted all of these genes, resulting in a collection of 57 candidate genes. We used them as ‘known’ driver genes for LUAD in the evaluation of our method.

#### Melanoma mutation data

The second mutation dataset was obtained from a recent large-scale NGS study of melanoma patients [16] (Text S1). WES was successfully performed in 121 tumor/normal pairs, with mutation data available for coding regions. In the original work, the authors highlighted 6 novel melanoma genes (*PPP6C*, *RAC1*, *SNX31*, *TACC1*, *STK19*, and *ARID2*).

#### Mutation annotation

We utilized the software tool ANNOVAR [33] and related annotation files to perform biological and functional annotations of these mutations. Only mutations in coding regions were considered, as they are more likely to be clinically relevant. To assess the functional impact of these mutations, we incorporated two popular prediction systems, SIFT [34] and PolyPhen2 [35], both of which are available through the ANNOVAR website. For the SIFT program, a lower score indicates a stronger probability to be deleterious. In contrast, a higher PolyPhen2 score indicates a stronger probability. In this work, we denoted a non-synonymous SNV to be deleterious if it has a SIFT score<0.05 or a PolyPhen2 score≥0.5. For indels in the coding regions, we denoted all of them as deleterious. In summary, our individual-based ‘deleterious mutation’ profile includes deleterious missense SNVs, all the other non-silent SNVs (nonsense, nonstop, splicing sites, and translation start sites), all non-silent dinucleotide polymorphisms (DNPs), all non-silent tri-nucleotide polymorphisms (TNPs), and all indels. The details of these mutations and filtering processes are provided in Figure S3. A mutation gene is denoted as a ‘MutGene’ if it harbors at least one deleterious mutation.

#### PPI and gene annotation data

We retrieved the most recent version of PPI data from the Human Protein Reference Database (release 9, 06/29/2010) [36] to serve as our reference network. Only the binary interactions were used. As a result, the complete PPI network included a total of 9617 proteins and 39,240 interactions.

We utilized the CCDS genes [37] (accessed 10/09/2012) to serve as a benchmark gene resource. All data used in this work, including both mutation data and network data, were mapped to CCDS genes. Only those that have matched CCDS gene symbols were retained for the follow-up analysis. For each CCDS gene, we estimated its cDNA length based on its coding sequences.

### Prioritization of mutation genes using Random Walk with Restart algorithm

Figure 1 shows the workflow, which has the following four steps.

Step 1. Patient-specific assessment of MutGenes. The aim of this step is to filter out potential genes whose mutations likely occur by chance based on the patient- (or sample-) specific mutational profile. Note the data and model fitting in this step are both performed for each single sample. As aforementioned, the likelihood of a gene to be mutated in a sample relies on many factors, including both genetic and environmental factors, which makes it impractical to accurately estimate the mutation rate for each gene. Here, instead of a direct estimation, we tackled this problem by formulating a generalized additive model and estimated a relative mutation rate for each gene. Given a cancer sample with MutGenes, let the vector *Y* denote the mutation status of each CCDS gene, i.e., *y_i_* = 1 if the *i* ^th^ gene is a MutGene in the sample and *y_i_* = 0 if it is not. A vector of *X* represents cDNA gene length. We formulate the following model to estimate the probability of a gene to be mutated as a function of its cDNA length, i.e.,
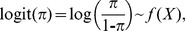
where *π* is the proportion of MutGenes in the investigated samples (i.e., 
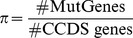
) and *f*(.) represents an unspecified smooth function. The function is then solved using a monotonic cubic spline with six knots. Based on the successful fitting of the function, each gene is assigned a weight, which represents its relative probability to be a MutGene (hereafter denoted as probability weight vector, or PWV) and is used as the gene-specific weight in the follow-up weighted resampling process. PWV retains the relative weight of each gene in a particular patient genome and this relative weight changes in different samples.

We then resampled random gene sets to build the null distribution of MutGenes occurring at random. Each random gene set has the same number of MutGenes. In this random selection procedure, each gene was selected from the genome following its probability weight as defined in the sample-specific PWV. The resampling process thus resembles the way in which MutGenes occur in a specific genome in random cases. The weighted resampling process was performed 1000 times in each sample, and a mutation frequency was computed for each gene using 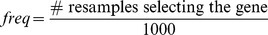. Here, a *freq*≥5% indicates the gene likely occurs at random and a frequency <5% indicates the gene is highly unlikely to be mutated due to random events. Accordingly, we filter genes with *freq*≥5%. Upon completion of this step, we obtained a list of significant MutGenes for each sample.

We attempt to fit sample-specific models using MutGenes for each sample such that the heterogeneous background of cancer patients can be properly considered. However, a practical challenge is to determine the minimum number of observations for reliable model fitting. For example, samples with very few MutGenes may not accomplish successful model fitting. Determination of the minimum number of observations remains an open question in statistics. In our case, to avoid arbitrary selection, we compared the results that were obtained using the sample-specific model with those obtained using the universal model. Here, the universal model was generated by using all MutGenes from the cohort. As shown in Figure S11, the difference in the retained MutGenes was large when samples had more MutGenes. We therefore selected 50 as the cutoff. For samples with ≥50 MutGenes [128 (70%) LUAD samples and 110 (91%) melanoma samples, Figure S4], we fitted a sample-specific model and obtained a sample-specific PWV. For other patients with fewer MutGenes, we performed a resample-based test using the universal PWV.

As a positive control, we examined the performance of the resample-based strategy on CGC genes, which are well-studied cancer genes. We found that 96.30% CGC genes had a frequency <5% in random datasets. Only 3.70% CGC genes had a frequency ≥5%. This result indicates our resample-based strategy retains a high sensitivity as evaluated by CGC genes; thus, the filtered genes are more likely randomly-occurring genes. Based on this observation, we created a manual adjustment to always retain CGC genes, even if they were occasionally observed with ≥5% frequency in random datasets. In practice, the users may remove this inclusion criterion.

Step 2. Sample-specific application of the Random Walk with Restart algorithm to search candidate interactors and MutGenes. The RWR algorithm simulates a random walker's transition in the network from a starting node (or a few starting nodes), with pre-defined starting probabilities, to its neighbors until it reaches a stable status. RWR allows for revisiting of the starting node(s) with revisiting probabilities. Given a network *G* with *n* nodes, we denote ***W*** as the column-normalized adjacency matrix for *G*; therefore, ***W*** is an *n*×*n* matrix. The RWR algorithm is formulated as:
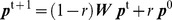
where *r* is the restart probability (e.g., *r* = 0.5 in this study), and ***p*** ^0^, ***p*** ^t^, and ***p*** ^t+1^ are vectors of size *n*. Each of the three parameters, ***p*** ^0^, ***p*** ^t^, and ***p*** ^t+1^, represents a vector in which the *i* ^th^ element holds the probability that the walker is at node *i* at time steps 0, *t*, and *t*+1, respectively. In general, assuming that there are *k* initial genes from which the walker would start with equal probability, the initial vector ***p*** ^0^ is defined as a vector, with the initial nodes having a probability of 1/*k* and the remaining nodes having a probability 0, such that the sum of the probabilities equals 1, i.e., 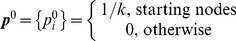, where *i* = 1,…,*n*. The RWR function is solved using this iteration process when the difference between ***p*** ^t^ and ***p*** ^t+1^ is below a predefined threshold (e.g., 10^−6^ in our analyses).

In each patient, we iteratively took each MutGene as the starting point to initiate the random walk and retained the top 1% (i.e., 10) of nodes that have the highest probabilities with which the walker would stay at a stable status as the highly accessible nodes for the initial node. Previous studies suggested various ways to select candidate nodes, e.g., the most accessible node (i.e., top 1) [18], top 5 [38], top 10 [39], [40], top 20 [40], and top 100 [41], but no consensus rules have been made. In this work, we chose to retain the top 10 accessible nodes. Although this selection criterion is arbitrary, our strategy is based on the observation that, in real biological networks, especially PPI networks, each node often has more than one important interactor. For example, TP53 is inhibited by the protein MDM2, but it is activated by ATM, both of which have a direct interaction with TP53 [36]. In such cases, consideration of only the most accessible interactor would overlook other important interactors. Taken together, the number of candidate interactors should not be too small (e.g., 1), as it may miss many important interactors; however, it should not be too large either, as many irrelevant genes may be included. We tested the selection of the top 1, top 5, and top 10 interactors using the data in this study. Based on the assessment, we selected 10 as a balance between choosing too few informative genes (e.g., top 1) and too many genes. However, this criterion can be adjusted depending on the specific data. It is worth noting that these 10 nodes (genes) that are most highly accessible from the starting node (gene) may not always be statistically significant compared to mere chance and will be evaluated in the next step.

Step 3. Randomization-based evaluation of the candidate interactors. To evaluate whether the subnetworks generated by RWR in step 2 do not occur by chance, we generated 100 random networks, each of which maintains the topological characteristics of the original network (e.g., degree of each node). We adapted the switching algorithm proposed by Milo et al. [42], which starts from the observed network and preserves the degree distribution in the generated random network.

We also performed RWR for MutGenes in each of the 100 random networks and we extracted the top 10 nodes with the highest probabilities. For each node encoded by a MutGene, the 10 candidate interactors in the observed network, *g_1_*, *g_2_*,…, *g_10_*, were assessed by computing an empirical *p*-value: 
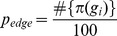
, where *π*(*g_i_*) is a random network in which *g_i_*, *i* = 1,…,10, was found as the top 10 candidate genes to the same initial node. The empirical *p*-value indicates the probability of a candidate interactor to be selected by chance. The interactors with *p*_edge_<0.05 are retained and denoted as significant interactors for the MutGene (see Figure 1).

Step 4. Construction of a consensus mutation subnetwork. After detecting MutGenes and their interactors in each sample, all significant interactions were pooled together, forming a universal candidate pool. This pool enabled us to better incorporate the information across multiple samples. After tabulating all edges, we explored the number of edges versus the edge occurrence (Figure 3). By fitting a linear regression model, we observed that the number of high frequency edges occurred more often than expected. A cutoff was selected according to the distribution (e.g., 10 for melanoma) and was manually adjusted based on expertise (e.g., 10 for LUAD) when necessary. Furthermore, we required both proteins involved in an interaction to be encoded by MutGenes. After pooling all the sample-specific MutGenes and their interactions, we implemented this step such that a pair of MutGenes and its interactor could be either mutated in the same patient or in different patients. In either instance, the interaction would be interrupted.

Next, we defined a parameter called the mutation recurrence (*mr*) index for each gene, or a pair of genes whose proteins interact, to control the false positive rate. The *mr* index is defined as 
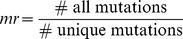
, where ‘# all mutations’ refers to mutations occurring in the gene across all samples, and ‘# unique mutations’ refers to the non-redundant set of ‘all mutations.’ Redundancy was determined if two mutations shared the same genomic coordinate regardless of the derived alleles. The introduction of the *mr* index is based on the observation that mutations in driver genes typically occur in important domains (e.g., kinase domains) and tend to cluster around ‘hotspots’ [43]. In contrast, mutations in passenger genes do not have particular features and may occur randomly across the whole gene. We removed interactions involving MutGenes whose *mr*<1.05. This cutoff of *mr* (<1.05) corresponds to MutGenes with >20 non-silent deleterious mutations in the cohort but none shared with any other (i.e., all are unique mutations). This filtering procedure resulted in a pool of high confidence interactions. Then, a consensus mutation network that was frequently mutated or revisited across many samples was derived by selecting the highly recurring interactions according to the overall distribution of the interaction pool.

### Functional enrichment analyses

We used the online tools DAVID [23] and ToppGene [44] for functional analyses. Both tools provide comprehensive resources for biological pathway annotation (e.g., canonical pathways from KEGG [24]) and biological processes (e.g., GO [25] terms). ToppGene also collected information from other databases, including BioCarta, BioCyc, Reactome, GenMAPP, and MSigDB. Wherever applicable, multiple testing correction using the Bonferroni method was performed to control the false discovery rate.

## Supporting Information

Figure S1 **Performance evaluation of four factors in VarWalker using lung adenocarcinoma samples.** (1) All MutGenes (denoted as “all”) versus recurrent MutGenes (“recurrent”). (2) The reference network: HPRD, PINA, and a network based on functional pathway annotation (denoted as “PathNet”). (3) Measurement of cDNA length: the actual cDNA length (“cDNA”) versus the sum of all possible non-silent mutations occurring in the cDNA regions (“NScount”). (4) Implementation of filtering genes that are two steps away from CGC genes (denoted as “cgcYes”) versus avoiding this filtering step (“cgcNo”).
(TIF)

Figure S2 **The proportion of genes retained after each step.** Three steps are examined: remove genes that failed gene length assessment by GAM (abbreviated as “By GAM”), map genes to the HPRD network (“Mapped to HPRD”), and remove genes that are two steps away from the CGC genes (“By step = 2 from CGC”).
(TIF)

Figure S3 **Somatic mutation profile for 183 lung adenocarcinoma (LUAD) samples (A) and 121 melanoma samples (B).** We retrieved raw mutation data from the supplemental information provided by the original publications [15], [16]. The mutations in LUAD samples were all somatic coding mutations [15]. Somatic mutations in the melanoma samples were provided for the whole gene regions (coding and noncoding) [16]. As described in the main text, deleterious mutations are denoted using grey boxes. The numbers shown in this figure include known SNPs from dbSNP or The 1000 Genomes Project. In our follow-up analyses, genes related to the mutations indicated in the grey boxes were further filtered by excluding known SNPs (dbSNP) according to the dbSNP_Val_Status in the original files.
(TIF)

Figure S4 **Distribution of MutGenes per sample.** The black vertical bars indicate the number of all MutGenes in each sample, and the red bars indicate the number of recurrent MutGenes in each sample.
(TIF)

Figure S5 **Distribution of genes' cDNA length (bp) in log_10_ scale.** All human CCDS genes were included. The 10 genes shown on the X-axis are the 10 most frequently mutated genes in the LUAD samples. *TP53* and *KRAS*, two well-known driver genes in LUAD, are shown in red.
(TIF)

Figure S6 **Evaluation of overlapping genes in independent datasets.** The null distribution of overlapping genes compared to those observed in lung adenocarcinoma and melanoma are plotted, respectively. The red dots in both panels indicate the observed number of overlapping genes.
(TIF)

Figure S7 **Consensus mutation network in lung adenocarcinoma (LUAD) samples.** Node size is proportional to the number of samples harboring mutations in the corresponding gene (MutGene), as indicated in the parenthesis following the node name. The triangular nodes denote the proteins encoded by known LUAD genes (see Materials and Methods). Edge width is proportional to the number of samples in which the interaction was observed, which is also indicated by the number on each edge. Note that only edges occurring in ≥10 samples are shown in the figure.
(TIF)

Figure S8 **Consensus mutation networks for LUAD smokers and never smokers.** (A) Distribution of the number of edges (in a logarithmic scale) versus their occurrence in LUAD smokers. (B) Consensus mutation networks for smokers. (C) Distribution of the number of edges (in a logarithmic scale) versus their occurrence in LUAD never smokers. (D) Consensus mutation networks for never smokers. In (B) and (D), node size is proportional to the number of samples harboring mutations in the corresponding gene (MutGene), as indicated in the parentheses after the node name. Note the node size is not in the same scale in (B) and (D) because there are only 27 never smokers and the mutation frequency is low. Edge width is proportional to the number of samples in which the interaction is detected, which is also indicated by the number on each edge.
(TIF)

Figure S9 **Consensus mutation network in melanoma samples.** Node size is proportional to the number of samples harboring mutations in the corresponding gene (MutGene), as indicated in the parenthesis after the node name. Edge width is proportional to the number of samples in which the interaction was observed, which is also indicated by the number on each edge. Note that only edges occurring in ≥10 samples are shown in the figure.
(TIF)

Figure S10 **Venn diagram of genes in the lung adenocarcinoma consensus mutation network, the melanoma consensus mutation network, and CGC genes.**
(TIF)

Figure S11 **Differences of the number of retained MutGenes after weighted resampling using the sample-specific probability weight vector (PWV) from that using universal PWV.** Y-axis: the difference between the number of retained genes when using the sample-specific PWV and that when using the universal PWV.
(TIF)

Table S1 **Comparison of data in the discovery and evaluation datasets for lung adenocarcinoma (LUAD) and melanoma.**
(DOCX)

Table S2 **Functional analysis of the mutation network for lung adenocarcinoma: Top significant KEGG pathways (** ***p*** **_Bonferroni_<10^−6^).**
(DOCX)

Table S3 **Functional analysis of the mutation network for lung adenocarcinoma: Top significant Gene Ontology (GO) terms in the Biology Process (BP) category (listed are** ***p*** **_Bonferroni_<10^−10^).**
(DOCX)

Table S4 **Functional analysis of the first subgraph in the mutation network for lung adenocarcinoma: top significant pathways.**
(DOCX)

Table S5 **Functional analysis of the second subgraph in the mutation network for lung adenocarcinoma (LUAD): Top 10 significant Gene Ontology (GO) terms in the Molecular Function (MF) and Biological Process (BP) categories.**
(DOCX)

Table S6 **Functional analysis of the third subgraph in the mutation network for lung adenocarcinoma (LUAD): Top 10 significant Gene Ontology (GO) terms in the Molecular Function (MF) and Biological Process (BP) categories.**
(DOCX)

Table S7 **Significant interactions in which both interactors are encoded by genes from known LUAD genes, Cancer Gene Census (CGC), or kinase and involve one highly mutated gene and one rarely mutated gene (in bold), as determined by the threshold for mutation frequency, i.e., 182×5% = 9.1 samples.**
(DOCX)

Table S8 **Functional analysis of the melanoma mutation network: Top significant KEGG pathways (** ***p*** **_Bonferroni_<10^−6^).**
(DOCX)

Table S9 **Functional analysis of the melanoma mutation network: Top significant GO terms (** ***p*** **_Bonferroni_<10^−6^).**
(DOCX)

Table S10 **Functional analysis of selected subgraphs in the melanoma mutation network: Top 10 significant pathways.**
(DOCX)

Table S11 **Functional analysis of 94 overlapping genes between the lung adenocarcinoma consensus mutation network and the melanoma consensus mutation network (top 5 in each category).**
(DOCX)

Text S1 **Detailed description of datasets and evaluation of the algorithm.**
(DOCX)
