# Supplementary figures and images for: VarWalker: Personalized Mutation Network Analysis of Putative Cancer Genes from Next-Generation Sequencing Data
Source: PLoS Comput Biol. 2014 Feb 6;10(2):e1003460. doi: 10.1371/journal.pcbi.1003460 (PMC3916227; doi:10.1371/journal.pcbi.1003460)

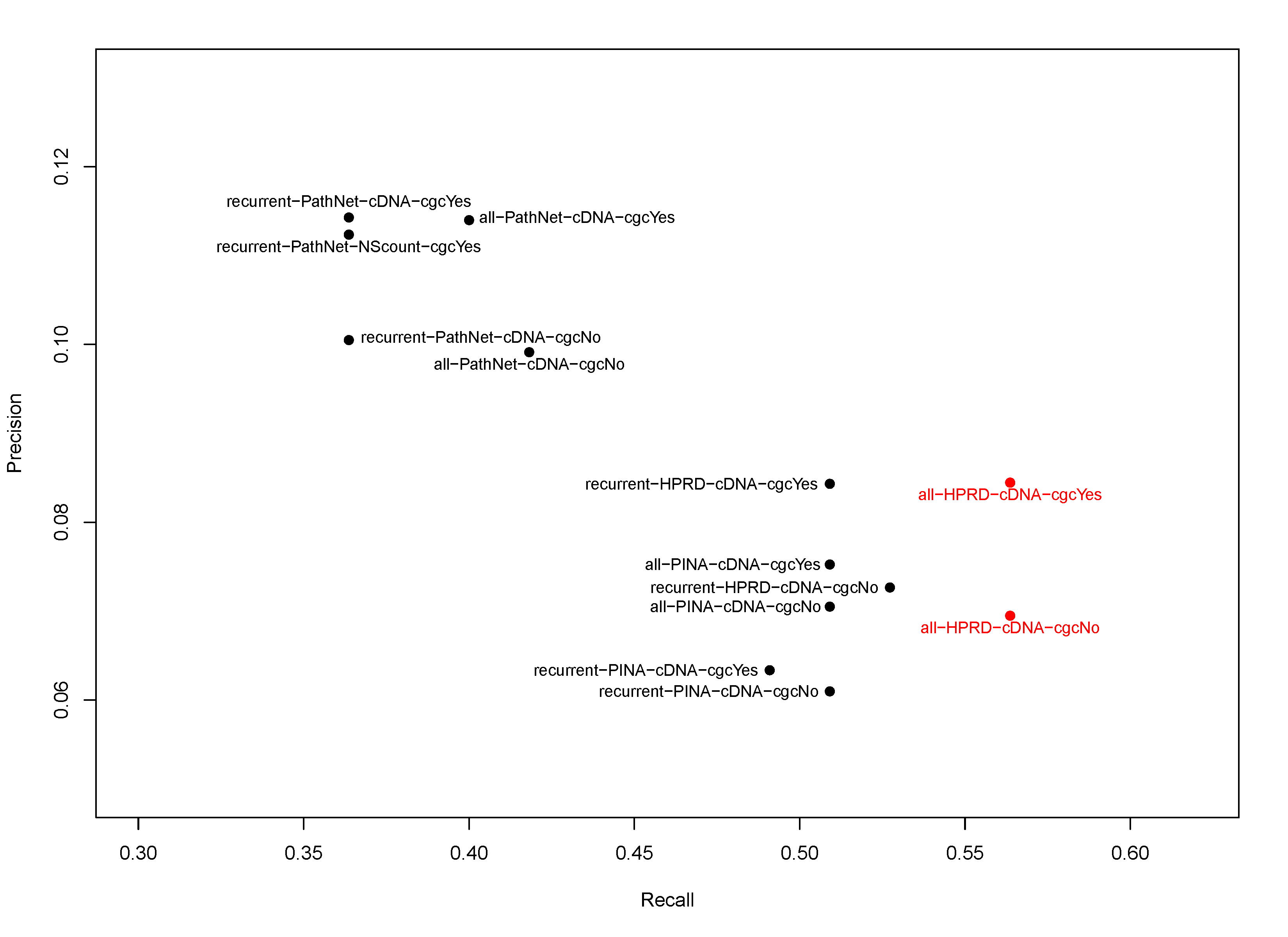

Supplement: Figure S1 — Performance evaluation of four factors in VarWalker using lung adenocarcinoma samples. (1) All MutGenes (denoted as “all”) versus recurrent MutGenes (“recurrent”). (2) The reference network: HPRD, PINA, and a network based on functional pathway annotation (denoted as “PathNet”). (3) Measurement of cDNA length: the actual cDNA length (“cDNA”) versus the sum of all possible non-silent mutations occurring in the cDNA regions (“NScount”). (4) Implementation of filtering genes that are two steps away from CGC genes (denoted as “cgcYes”) versus avoiding this filtering step (“cgcNo”). (TIF) [file pcbi.1003460.s001.tif]

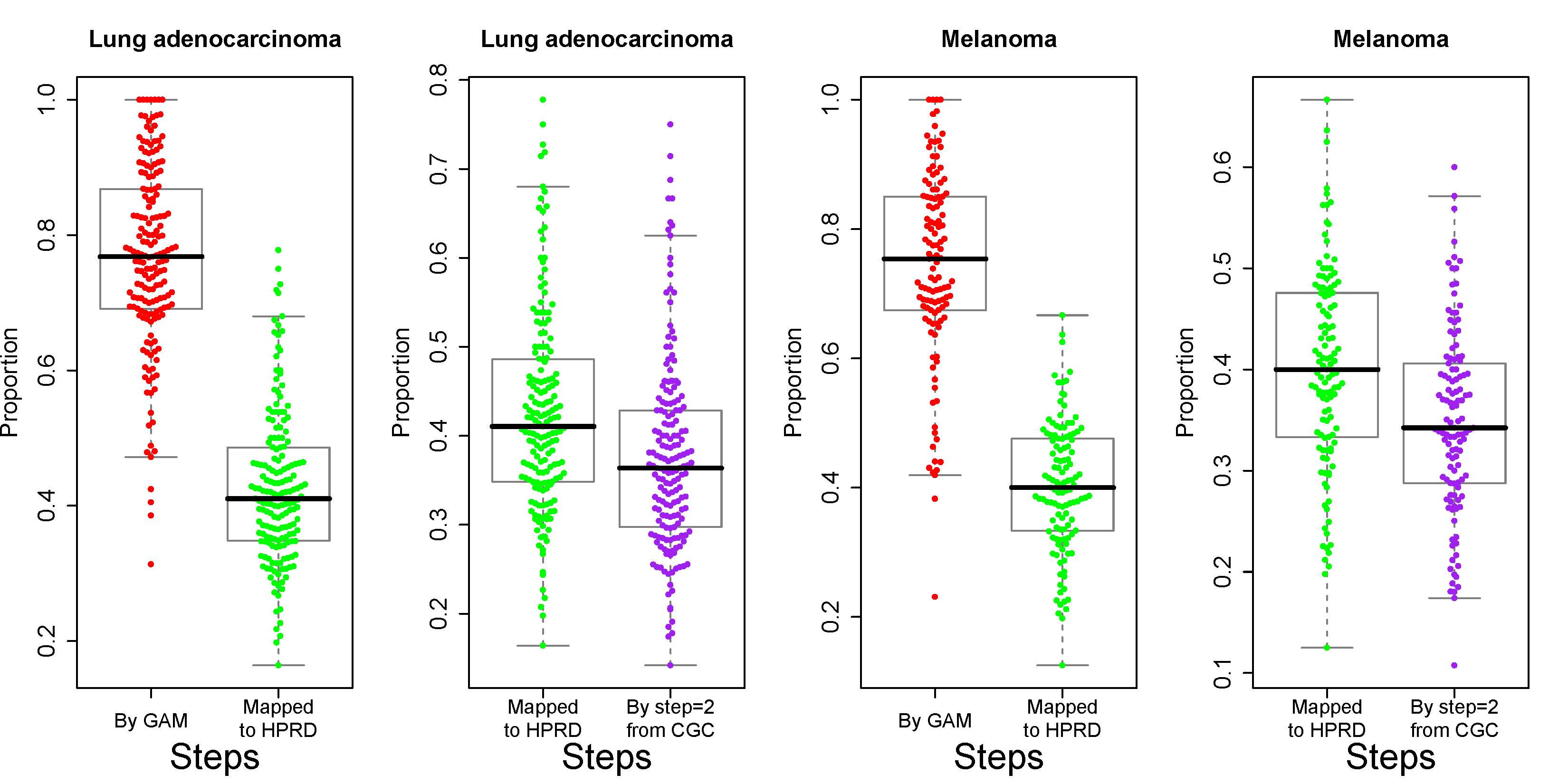

Supplement: Figure S2 — The proportion of genes retained after each step. Three steps are examined: remove genes that failed gene length assessment by GAM (abbreviated as “By GAM”), map genes to the HPRD network (“Mapped to HPRD”), and remove genes that are two steps away from the CGC genes (“By step = 2 from CGC”). (TIF) [file pcbi.1003460.s002.tif]

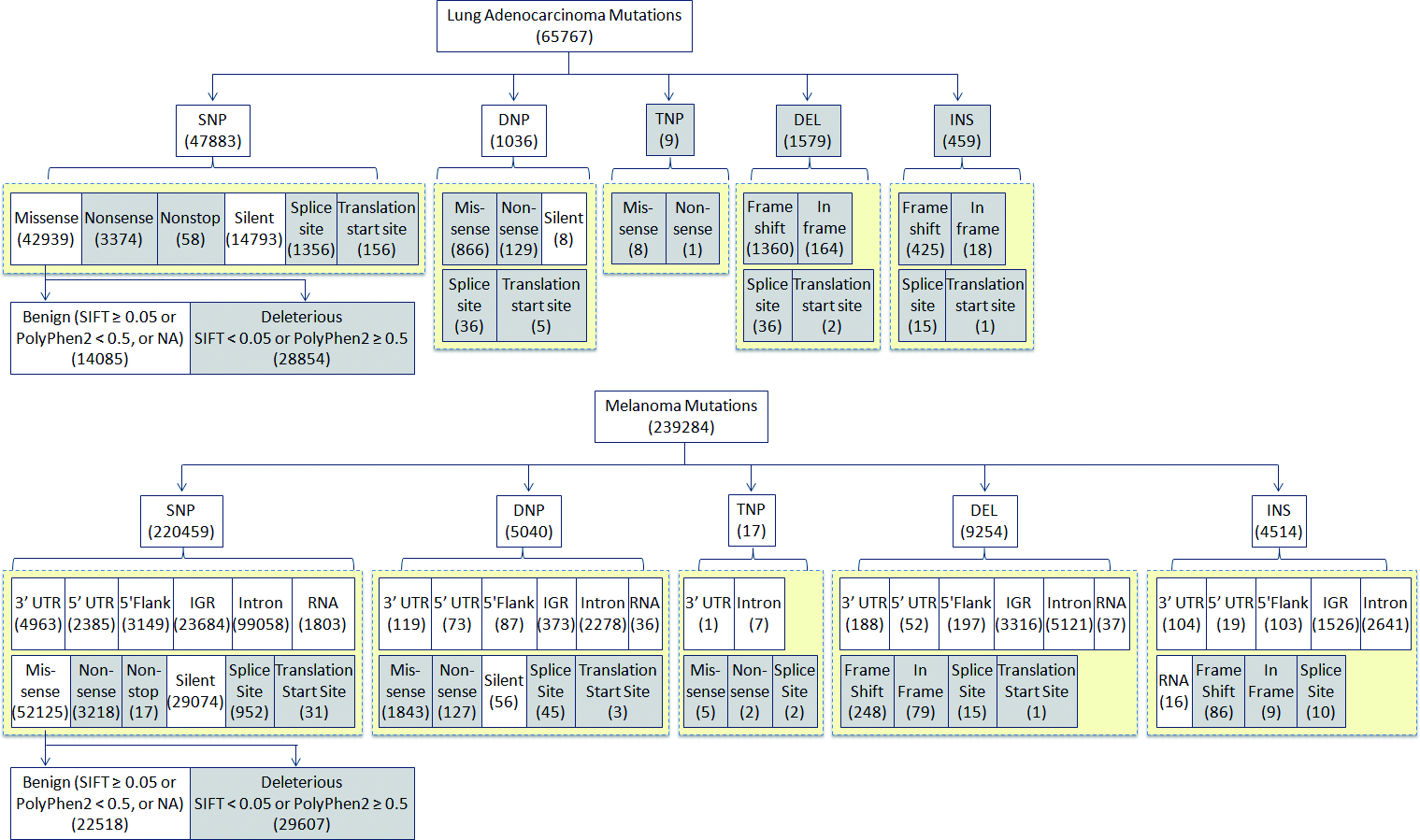

Supplement: Figure S3 — Somatic mutation profile for 183 lung adenocarcinoma (LUAD) samples (A) and 121 melanoma samples (B). We retrieved raw mutation data from the supplemental information provided by the original publications [15], [16]. The mutations in LUAD samples were all somatic coding mutations [15]. Somatic mutations in the melanoma samples were provided for the whole gene regions (coding and noncoding) [16]. As described in the main text, deleterious mutations are denoted using grey boxes. The numbers shown in this figure include known SNPs from dbSNP or The 1000 Genomes Project. In our follow-up analyses, genes related to the mutations indicated in the grey boxes were further filtered by excluding known SNPs (dbSNP) according to the dbSNP_Val_Status in the original files. (TIF) [file pcbi.1003460.s003.tif]

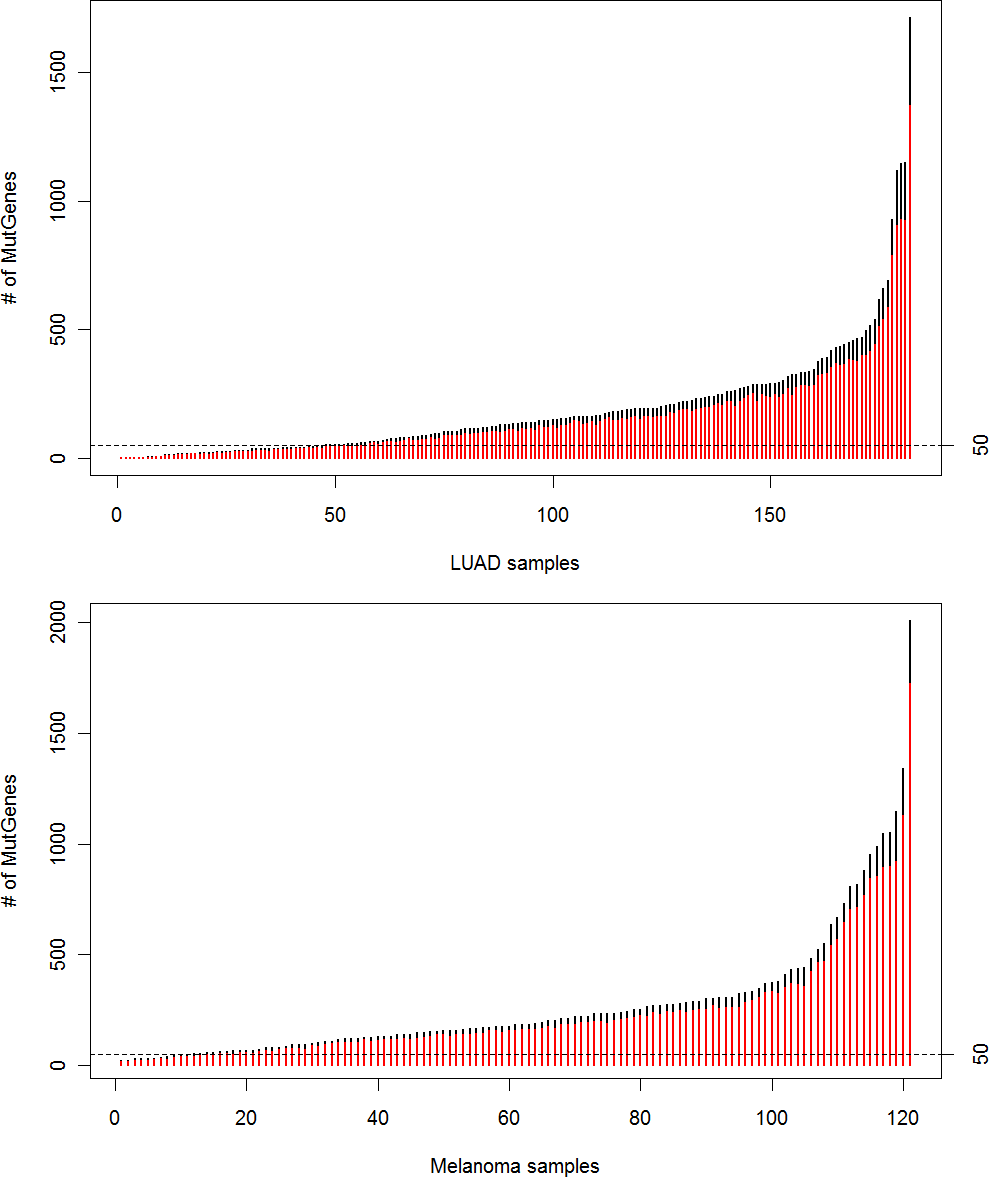

Supplement: Figure S4 — Distribution of MutGenes per sample. The black vertical bars indicate the number of all MutGenes in each sample, and the red bars indicate the number of recurrent MutGenes in each sample. (TIF) [file pcbi.1003460.s004.tif]

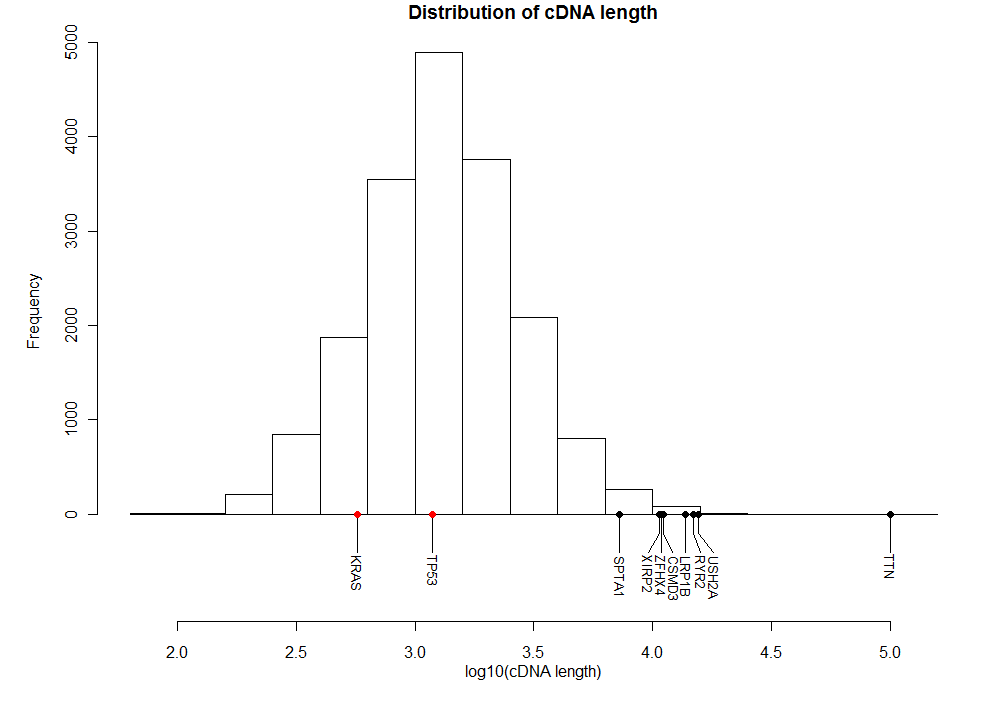

Supplement: Figure S5 — Distribution of genes' cDNA length (bp) in log10 scale. All human CCDS genes were included. The 10 genes shown on the X-axis are the 10 most frequently mutated genes in the LUAD samples. TP53 and KRAS, two well-known driver genes in LUAD, are shown in red. (TIF) [file pcbi.1003460.s005.tif]

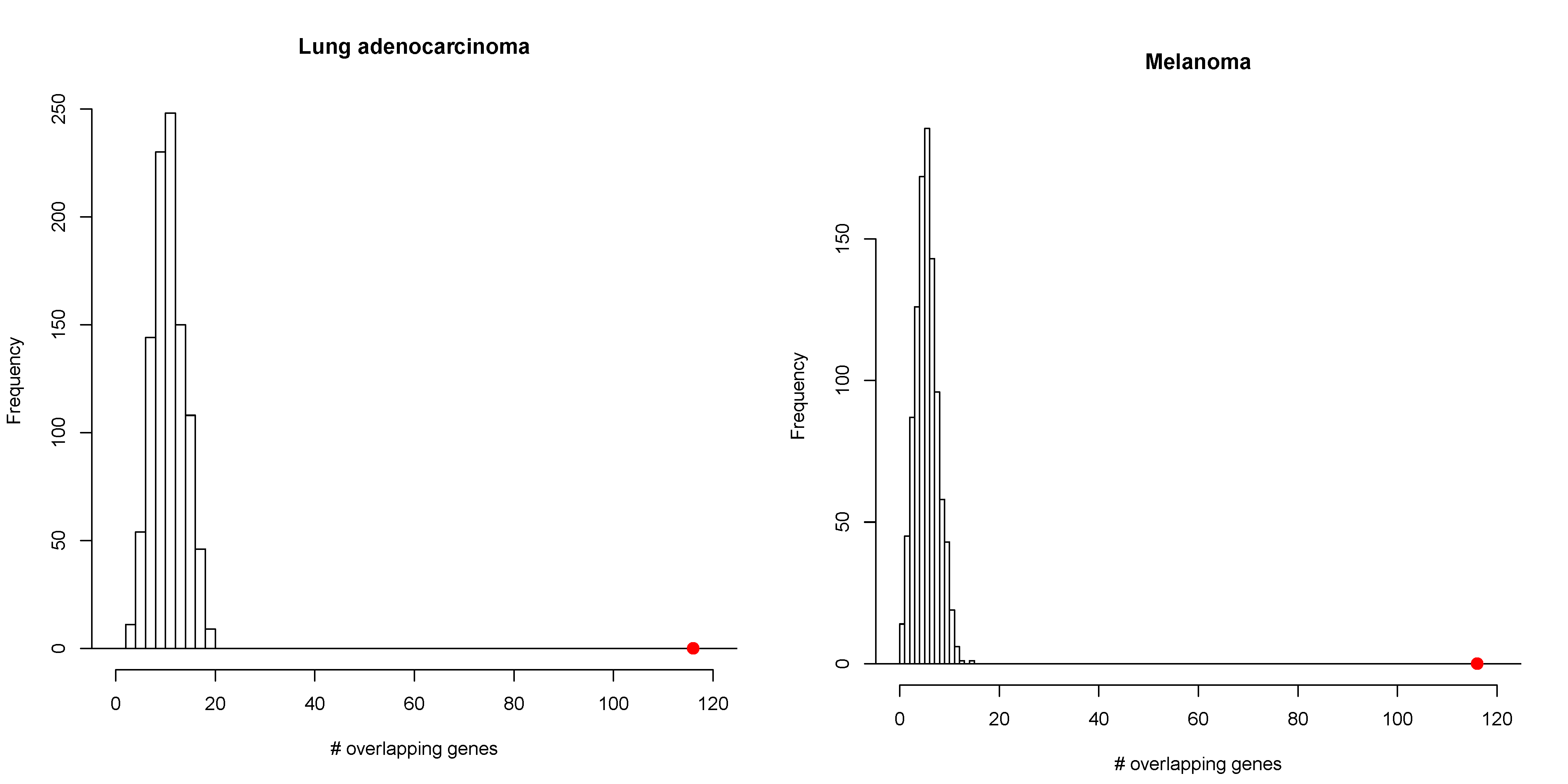

Supplement: Figure S6 — Evaluation of overlapping genes in independent datasets. The null distribution of overlapping genes compared to those observed in lung adenocarcinoma and melanoma are plotted, respectively. The red dots in both panels indicate the observed number of overlapping genes. (TIF) [file pcbi.1003460.s006.tif]

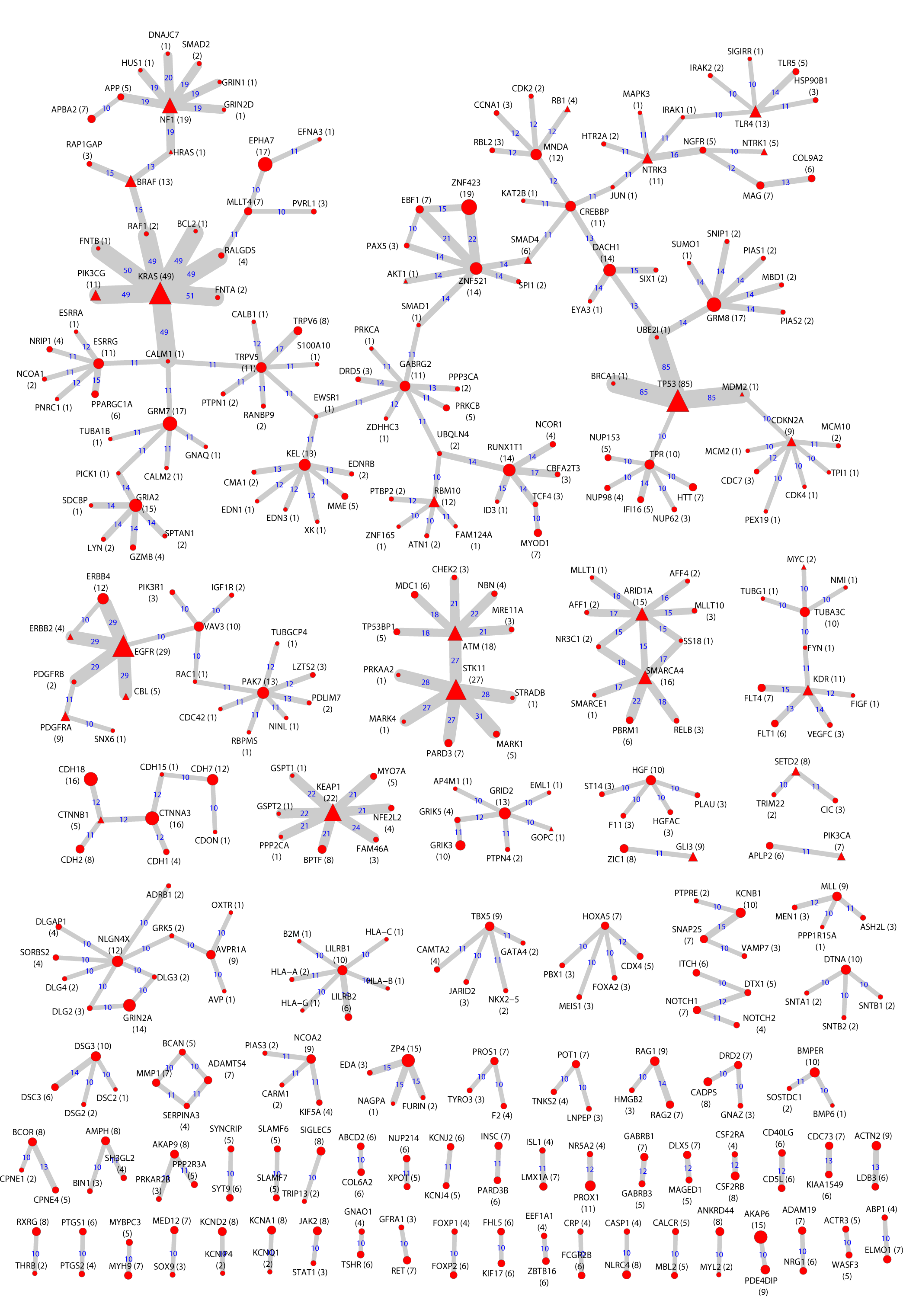

Supplement: Figure S7 — Consensus mutation network in lung adenocarcinoma (LUAD) samples. Node size is proportional to the number of samples harboring mutations in the corresponding gene (MutGene), as indicated in the parenthesis following the node name. The triangular nodes denote the proteins encoded by known LUAD genes (see Materials and Methods). Edge width is proportional to the number of samples in which the interaction was observed, which is also indicated by the number on each edge. Note that only edges occurring in ≥10 samples are shown in the figure. (TIF) [file pcbi.1003460.s007.tif]

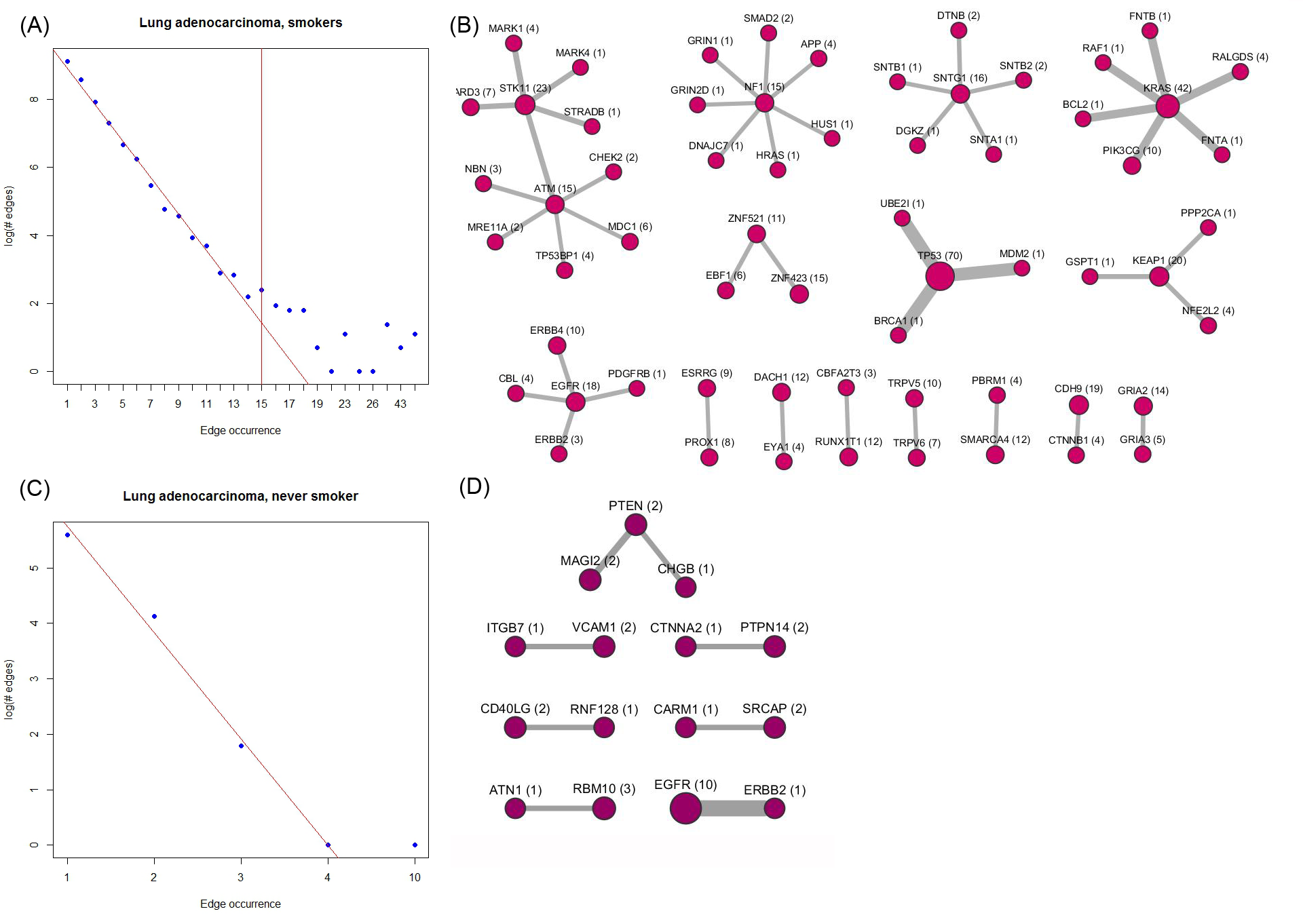

Supplement: Figure S8 — Consensus mutation networks for LUAD smokers and never smokers. (A) Distribution of the number of edges (in a logarithmic scale) versus their occurrence in LUAD smokers. (B) Consensus mutation networks for smokers. (C) Distribution of the number of edges (in a logarithmic scale) versus their occurrence in LUAD never smokers. (D) Consensus mutation networks for never smokers. In (B) and (D), node size is proportional to the number of samples harboring mutations in the corresponding gene (MutGene), as indicated in the parentheses after the node name. Note the node size is not in the same scale in (B) and (D) because there are only 27 never smokers and the mutation frequency is low. Edge width is proportional to the number of samples in which the interaction is detected, which is also indicated by the number on each edge. (TIF) [file pcbi.1003460.s008.tif]

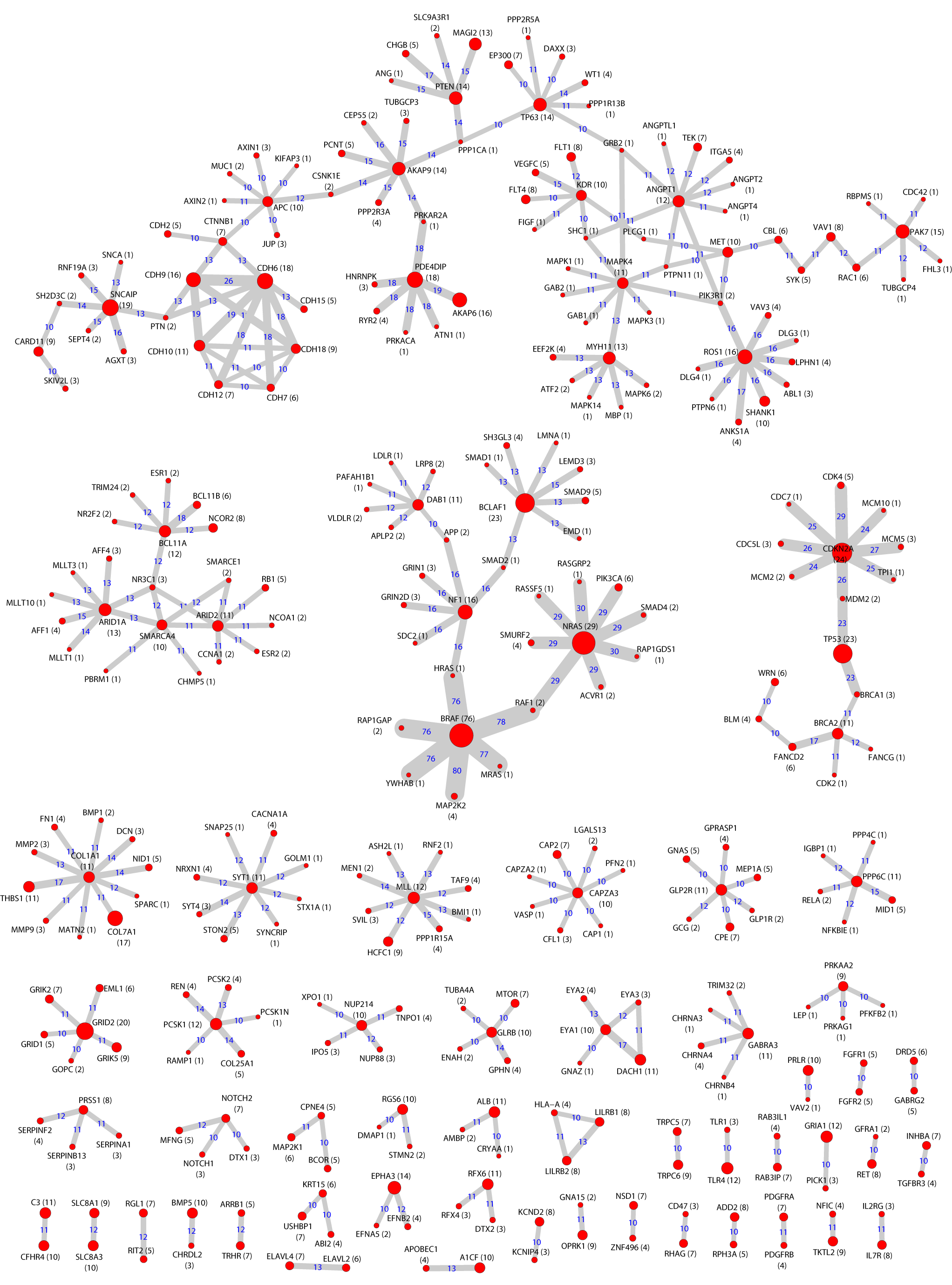

Supplement: Figure S9 — Consensus mutation network in melanoma samples. Node size is proportional to the number of samples harboring mutations in the corresponding gene (MutGene), as indicated in the parenthesis after the node name. Edge width is proportional to the number of samples in which the interaction was observed, which is also indicated by the number on each edge. Note that only edges occurring in ≥10 samples are shown in the figure. (TIF) [file pcbi.1003460.s009.tif]

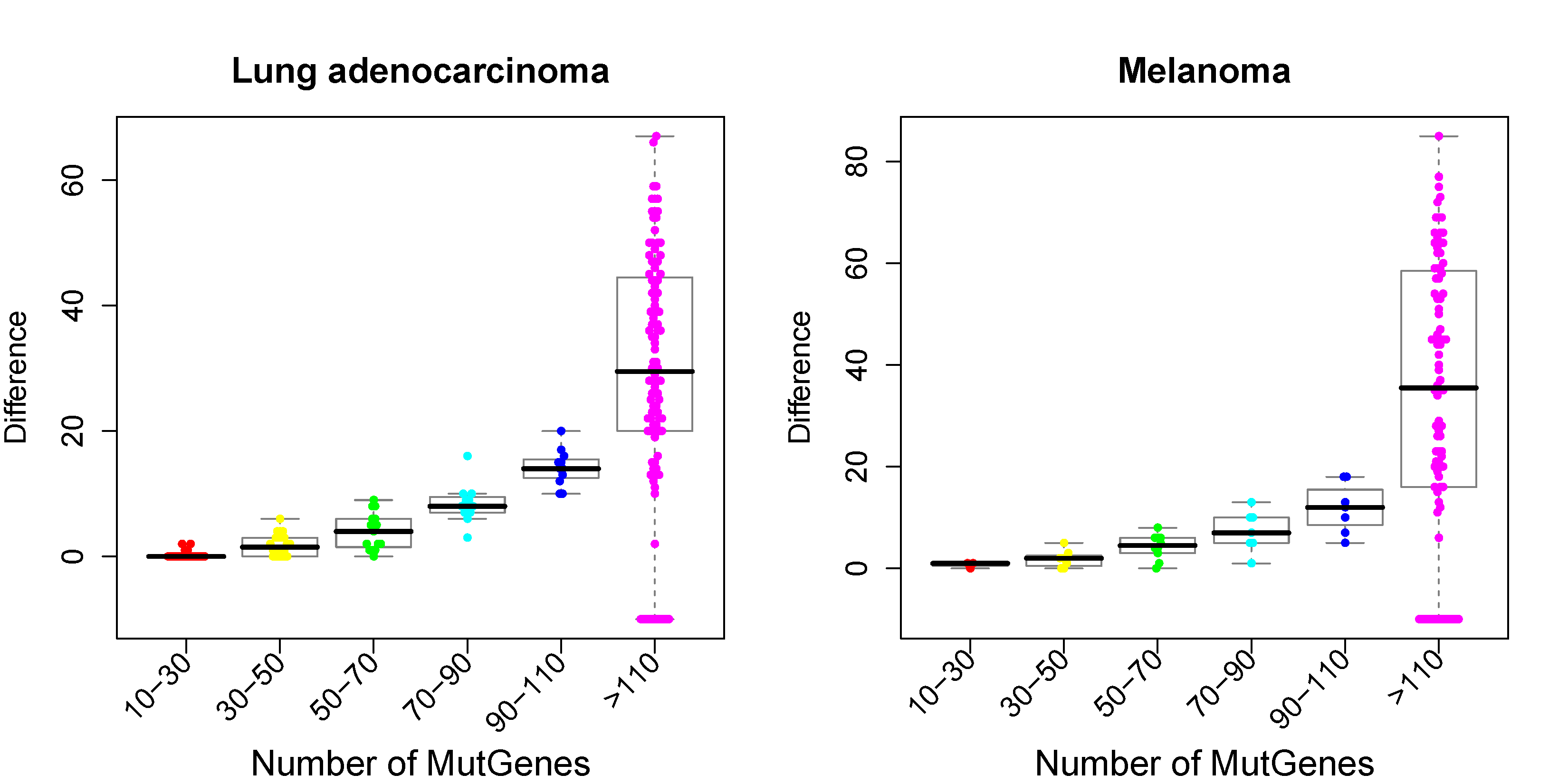

Supplement: Figure S11 — Differences of the number of retained MutGenes after weighted resampling using the sample-specific probability weight vector (PWV) from that using universal PWV. Y-axis: the difference between the number of retained genes when using the sample-specific PWV and that when using the universal PWV. (TIF) [file pcbi.1003460.s011.tif]
