# Supplementary material for: VarWalker: Personalized Mutation Network Analysis of Putative Cancer Genes from Next-Generation Sequencing Data
Source: PLoS Comput Biol. 2014 Feb 6;10(2):e1003460. doi: 10.1371/journal.pcbi.1003460 (PMC3916227; doi:10.1371/journal.pcbi.1003460)
Supplement: Table S1 — Comparison of data in the discovery and evaluation datasets for lung adenocarcinoma (LUAD) and melanoma. (DOCX) [file pcbi.1003460.s012.docx]

**Table S1**. Comparison of data in the discovery and evaluation datasets for lung adenocarcinoma (LUAD) and melanoma.

|  | Lung adenocarcinoma | | | Melanoma | | |
| --- | --- | --- | --- | --- | --- | --- |
|  | Discovery | Evaluation | | Discovery | Evaluation | |
| Dataset name | LUAD | TCGA_LUAD | | Melanoma | TCGA_SKCM | |
| Reference | Imielinski et al. 2012 | Unpublished | | Hodis et al. 2012 | Unpublished | |
| # samples | 182 | 518 (as of 7/18/2013) | | 121 | 264 (as of 8/19/2013) | |
| # MutGenes | 11,306 | 15,328 | | 11,030 | 15,028 | |
| # genes in the mutation network | 367 | 218 | | 331 | 117 | |
| # overlap genes | 1161 (31.6%) | | 116 (53.21%) | 86 (25.98%) | | 86 (73.50%) |
