# Supplementary material for: VarWalker: Personalized Mutation Network Analysis of Putative Cancer Genes from Next-Generation Sequencing Data
Source: PLoS Comput Biol. 2014 Feb 6;10(2):e1003460. doi: 10.1371/journal.pcbi.1003460 (PMC3916227; doi:10.1371/journal.pcbi.1003460)
Supplement: Table S2 — Functional analysis of the mutation network for lung adenocarcinoma: Top significant KEGG pathways ( p Bonferroni<10−6). (DOCX) [file pcbi.1003460.s013.docx]

**Table S2**. Functional analysis of the mutation network for lung adenocarcinoma: top significant KEGG pathways (*p*_Bonferroni_ <10^-6^).

| **KEGG pathway** | **# genes in the consensus mutation network** | ***p-*value^*^** | ***p*_Bonferroni_** |
| --- | --- | --- | --- |
| hsa05200:Pathways in cancer | 55 | 1.50×10^-22^ | 1.60×10^-20^ |
| hsa05214:Glioma | 21 | 2.53×10^-14^ | 2.71×10^-12^ |
| hsa05212:Pancreatic cancer | 22 | 3.71×10^-14^ | 3.97×10^-12^ |
| hsa05219:Bladder cancer | 17 | 4.26×10^-13^ | 4.56×10^-11^ |
| hsa05223:Non-small cell lung cancer | 18 | 2.86×10^-12^ | 3.06×10^-10^ |
| hsa05215:Prostate cancer | 22 | 3.63×10^-12^ | 3.89×10^-10^ |
| hsa05218:Melanoma | 20 | 3.65×10^-12^ | 3.91×10^-10^ |
| hsa04510:Focal adhesion | 32 | 3.80×10^-12^ | 4.07×10^-10^ |
| hsa05210:Colorectal cancer | 21 | 1.03×10^-11^ | 1.10×10^-9^ |
| hsa04520:Adherens junction | 19 | 1.64×10^-10^ | 1.75×10^-8^ |
| hsa05213:Endometrial cancer | 16 | 2.36×10^-10^ | 2.53×10^-8^ |
| hsa04012:ErbB signaling pathway | 19 | 1.40×10^-9^ | 1.49×10^-7^ |
| hsa05216:Thyroid cancer | 12 | 2.41×10^-9^ | 2.58×10^-7^ |
| hsa05211:Renal cell carcinoma | 17 | 2.56×10^-9^ | 2.74×10^-7^ |
| hsa05220:Chronic myeloid leukemia | 17 | 7.52×10^-9^ | 8.04×10^-7^ |

^*^ The *p*-value is a modified Fisher Exact *p*-value, i.e., EASE score as provided by DAVID (http://david.abcc.ncifcrf.gov/) [6]. The smaller the *p* value, the more MutGenes enriched.
