# Supplementary material for: VarWalker: Personalized Mutation Network Analysis of Putative Cancer Genes from Next-Generation Sequencing Data
Source: PLoS Comput Biol. 2014 Feb 6;10(2):e1003460. doi: 10.1371/journal.pcbi.1003460 (PMC3916227; doi:10.1371/journal.pcbi.1003460)
Supplement: Table S3 — Functional analysis of the mutation network for lung adenocarcinoma: Top significant Gene Ontology (GO) terms in the Biology Process (BP) category (listed are p Bonferroni<10−10). (DOCX) [file pcbi.1003460.s014.docx]

**Table S3**. Functional analysis of the mutation network for lung adenocarcinoma: top significant Gene Ontology (GO) terms in the Biology Process (BP) category (*p*_Bonferroni_ <10^-10^).

| **GO ID (BP)** | **GO term** | **Count** | ***p*-value** | ***p*_Bonferroni_** |
| --- | --- | --- | --- | --- |
| GO:0009891 | Positive regulation of biosynthetic process | 70 | 2.42×10^-23^ | 6.97×10^-20^ |
| GO:0042127 | Regulation of cell proliferation | 74 | 6.15×10^-23^ | 1.77×10^-19^ |
| GO:0051173 | Positive regulation of nitrogen compound metabolic process | 66 | 2.29×10^-22^ | 6.58×10^-19^ |
| GO:0031328 | Positive regulation of cellular biosynthetic process | 68 | 2.69×10^-22^ | 7.73×10^-19^ |
| GO:0010604 | Positive regulation of macromolecule metabolic process | 75 | 2.12×10^-21^ | 6.10×10^-18^ |
| GO:0010557 | Positive regulation of macromolecule biosynthetic process | 64 | 1.28×10^-20^ | 3.68×10^-17^ |
| GO:0045935 | Positive regulation of nucleobase, nucleoside, nucleotide and nucleic acid metabolic process | 62 | 2.73×10^-20^ | 7.86×10^-17^ |
| GO:0043067 | Regulation of programmed cell death | 66 | 4.08×10^-17^ | 1.17×10^-13^ |
| GO:0010941 | Regulation of cell death | 66 | 4.90×10^-17^ | 1.41×10^-13^ |
| GO:0045941 | Positive regulation of transcription | 54 | 6.11×10^-17^ | 3.19×10^-13^ |
| GO:0042981 | Regulation of apoptosis | 65 | 9.75×10^-17^ | 3.19×10^-13^ |
| GO:0010628 | Positive regulation of gene expression | 54 | 3.28×10^-16^ | 9.58×10^-13^ |
| GO:0008284 | Positive regulation of cell proliferation | 45 | 5.07×10^-16^ | 1.60×10^-12^ |
| GO:0045893 | Positive regulation of transcription, DNA-dependent | 48 | 9.08×10^-16^ | 2.55×10^-12^ |
| GO:0051254 | Positive regulation of RNA metabolic process | 48 | 1.17×10^-15^ | 3.51×10^-12^ |
| GO:0007242 | Intracellular signaling cascade | 80 | 1.32×10^-14^ | 3.80×10^-11^ |
| GO:0044057 | Regulation of system process | 37 | 1.63×10^-14^ | 4.69×10^-11^ |
