# Supplementary material for: VarWalker: Personalized Mutation Network Analysis of Putative Cancer Genes from Next-Generation Sequencing Data
Source: PLoS Comput Biol. 2014 Feb 6;10(2):e1003460. doi: 10.1371/journal.pcbi.1003460 (PMC3916227; doi:10.1371/journal.pcbi.1003460)
Supplement: Table S4 — Functional analysis of the first subgraph in the mutation network for lung adenocarcinoma: top significant pathways. (DOCX) [file pcbi.1003460.s015.docx]

**Table S4**. Functional analysis of the first subgraph in the mutation network for lung adenocarcinoma: top significant pathways.

| **Pathway** | **Source** | ***p*_Bonferroni_** | **Genes in the first subgraph** |
| --- | --- | --- | --- |
| WP2032: TSH signaling pathway | WikiPathways | 4.33×10^-9^ | *HRAS, RAP1GAP, RALGDS, RAF1, GNAQ, RBL2, RB1, JUN, BRAF, MAPK3, CDK2, AKT1, CDK4* |
| BIOCARTA: Signaling Pathway from G-Protein Families | MSigDB: C2.cp - BioCarta | 2.96×10^-8^ | *CALM2, HRAS, CALM1, RAF1, GNAQ, JUN, MAPK3, PRKCA, PRKCB, PPP3CA* |
| BIOCARTA: BCR Signaling Pathway | MSigDB: C2.cp - BioCarta | 2.96×10^-8^ | *LYN, CALM2, HRAS, CALM1, RAF1, JUN, MAPK3, PRKCA, PRKCB, PPP3CA* |
| BIOCARTA: Fc Epsilon Receptor I Signaling in Mast Cells | MSigDB: C2.cp - BioCarta | 9.77×10^-8^ | *LYN, CALM2, HRAS, CALM1, RAF1, JUN, MAPK3, PRKCB, PIK3CG, PPP3CA* |
| BIOCARTA: Links between Pyk2 and Map Kinases | MSigDB: C2.cp - BioCarta | 1.28×10^-7^ | *CALM2, HRAS, CALM1, RAF1, GNAQ, JUN, MAPK3, PRKCA, PRKCB* |
| WP138: Androgen receptor signaling pathway | WikiPathways | 1.53×10^-7^ | *SMAD4, CREBBP, PIAS1, MDM2, UBE2I, SUMO1, RB1, JUN, BRCA1, NCOA1, NCOR1, AKT1, KAT2B* |
| tcrraspathway: Ras signaling in the CD4+ TCR pathway | NCI-Nature Curated | 2.79×10^-7^ | *HRAS, RAF1, BRAF, MAPK3, PRKCA, PRKCB, KRAS* |
| BIOCARTA: Mechanism of Gene Regulation by Peroxisome Proliferators via PPARa(alpha) | MSigDB: C2.cp - BioCarta | 3.73×10^-7^ | *CREBBP, NRIP1, PPARGC1A, RB1, JUN, NCOA1, NCOR1, MAPK3, PRKCA, PRKCB, PIK3CG* |
| BIOCARTA: Angiotensin II mediated activation of JNK Pathway via Pyk2 dependent signaling | MSigDB: C2.cp - BioCarta | 6.33×10^-7^ | *CALM2, HRAS, CALM1, RAF1, GNAQ, JUN, MAPK3, PRKCA, PRKCB* |
| BIOCARTA: T Cell Receptor Signaling Pathway | MSigDB: C2.cp - BioCarta | 7.23×10^-7^ | *CALM2, HRAS, CALM1, RAF1, JUN, MAPK3, PRKCA, PRKCB, PIK3CG, PPP3CA* |
| **smad2_3nuclearpathway: Regulation of nuclear SMAD2/3 signaling** | **NCI-Nature Curated** | **1.17×10^-6^** | ***SMAD2, SMAD4, MYOD1, CREBBP, JUN, SNIP1, NCOA1, NCOR1, CDK2, AKT1, CDK4, KAT2B*** |
