# Supplementary material for: VarWalker: Personalized Mutation Network Analysis of Putative Cancer Genes from Next-Generation Sequencing Data
Source: PLoS Comput Biol. 2014 Feb 6;10(2):e1003460. doi: 10.1371/journal.pcbi.1003460 (PMC3916227; doi:10.1371/journal.pcbi.1003460)
Supplement: Table S5 — Functional analysis of the second subgraph in the mutation network for lung adenocarcinoma (LUAD): Top 10 significant Gene Ontology (GO) terms in the Molecular Function (MF) and Biological Process (BP) categories. (DOCX) [file pcbi.1003460.s016.docx]

**Table S5**. Functional analysis of the second subgraph in the mutation network for lung adenocarcinoma (LUAD): top 10 significant Gene Ontology (GO) terms in the Molecular Function (MF) and Biological Process (BP) categories.

| **GO term** | ***p*_Bonferroni_** | **Genes in the second subgraph** |
| --- | --- | --- |
| *Molecular Function* |  |  |
| **GO:0004714: transmembrane receptor protein tyrosine kinase activity** | **3.45×10^-9^** | ***ERBB2, ERBB4, PDGFRB, PDGFRA, EGFR, IGF1R*** |
| GO:0019199: transmembrane receptor protein kinase activity | 1.37×10^-8^ | *ERBB2, ERBB4, PDGFRB, PDGFRA, EGFR, IGF1R* |
| GO:0004713: protein tyrosine kinase activity | 4.32×10^-7^ | *ERBB2, ERBB4, PDGFRB, PDGFRA, EGFR, IGF1R* |
| GO:0043548: phosphatidylinositol 3-kinase binding | 1.06×10^-6^ | *PDGFRB, PIK3R1, PDGFRA, IGF1R* |
| **GO:0005102: receptor binding** | **6.39×10^-6^** | ***ERBB2, ERBB4, PDGFRB, VAV3, PIK3R1, PDGFRA, EGFR, CBL, IGF1R, CDC42*** |
| GO:0019838: growth factor binding | 7.60×10^-6^ | *ERBB2, PDGFRB, PDGFRA, EGFR, IGF1R* |
| GO:0070851: growth factor receptor binding | 9.79×10^-6^ | *ERBB4, PDGFRB, VAV3, PIK3R1, PDGFRA* |
| GO:0004716: receptor signaling protein tyrosine kinase activity | 1.14×10^-5^ | *ERBB2, ERBB4, EGFR* |
| GO:0016773: phosphotransferase activity, alcohol group as acceptor | 1.52×10^-5^ | *ERBB2, ERBB4, PDGFRB, PIK3R1, PDGFRA, EGFR, PAK7, IGF1R* |
| GO:0016301: kinase activity | 4.44×10^-5^ | *ERBB2, ERBB4, PDGFRB, PIK3R1, PDGFRA, EGFR, PAK7, IGF1R* |
| *Biological Process* |  |  |
| GO:0043551:regulation of phosphatidylinositol 3-kinase activity | 9.39×10^-13^ | *ERBB4, PDGFRB, VAV3, PIK3R1, PDGFRA, RAC1, CDC42* |
| GO:0043550:regulation of lipid kinase activity | 2.87×10^-12^ | *ERBB4, PDGFRB, VAV3, PIK3R1, PDGFRA, RAC1, CDC42* |
| GO:0007169:transmembrane receptor protein tyrosine kinase signaling pathway | 8.90×10^-11^ | *ERBB2, ERBB4, SNX6, PDGFRB, VAV3, PIK3R1, PDGFRA, EGFR, CBL, RAC1, IGF1R, CDC42* |
| GO:0043552:positive regulation of phosphatidylinositol 3-kinase activity | 1.27×10^-10^ | *ERBB4, PDGFRB, VAV3, PDGFRA, RAC1, CDC42* |
| GO:0007167:enzyme linked receptor protein signaling pathway | 1.36×10^-10^ | *ERBB2, ERBB4, SNX6,PDGFRB, VAV3, PIK3R1, PDGFRA, EGFR, RBPMS, CBL, RAC1, IGF1R, CDC42* |
| GO:0090218:positive regulation of lipid kinase activity | 1.62×10^-10^ | *ERBB4, PDGFRB, VAV3, PDGFRA, RAC1, CDC42* |
| GO:0042325:regulation of phosphorylation | 5.33×10^-9^ | *ERBB2, ERBB4, SNX6, PDGFRB, VAV3, PIK3R1, PDGFRA, EGFR, RBPMS, RAC1, IGF1R, CDC42* |
| GO:0043549:regulation of kinase activity | 5.54×10^-9^ | *ERBB2, ERBB4, SNX6, PDGFRB, VAV3, PIK3R1, PDGFRA, EGFR, RAC1, IGF1R, CDC42* |
| GO:0045834:positive regulation of lipid metabolic process | 7.48×10^-9^ | *ERBB4, PDGFRB, VAV3, PDGFRA, RAC1, IGF1R, CDC42* |
| GO:0051338:regulation of transferase activity | 7.68×10^-9^ | *ERBB2, ERBB4, SNX6, PDGFRB, VAV3, PIK3R1, PDGFRA, EGFR, RAC1, IGF1R, CDC42* |
| GO:0019216:regulation of lipid metabolic process | 3.90×10^-8^ | *ERBB4, PDGFRB, VAV3, PIK3R1, PDGFRA, RAC1, IGF1R, CDC42* |
