# Supplementary material for: VarWalker: Personalized Mutation Network Analysis of Putative Cancer Genes from Next-Generation Sequencing Data
Source: PLoS Comput Biol. 2014 Feb 6;10(2):e1003460. doi: 10.1371/journal.pcbi.1003460 (PMC3916227; doi:10.1371/journal.pcbi.1003460)
Supplement: Table S6 — Functional analysis of the third subgraph in the mutation network for lung adenocarcinoma (LUAD): Top 10 significant Gene Ontology (GO) terms in the Molecular Function (MF) and Biological Process (BP) categories. (DOCX) [file pcbi.1003460.s017.docx]

**Table S6**. Functional analysis of the third subgraph in the mutation network for lung adenocarcinoma (LUAD): top 10 significant Gene Ontology (GO) terms in the Molecular Function (MF) and Biological Process (BP) categories.

| **GO term** | ***p*_Bonferroni_** | **Genes in the third subgraph** |
| --- | --- | --- |
| *Molecular Function* |  |  |
| GO:0004672:protein kinase activity | 2.92×10^-6^ | *ATM, MARK1, CHEK2, PRKAA2, MARK4, STK11, STRADB* |
| GO:0016773:phosphotransferase activity, alcohol group as acceptor | 9.52×10^-6^ | *ATM, MARK1, CHEK2, PRKAA2, MARK4, STK11, STRADB* |
| GO:0004674:protein serine/threonine kinase activity | 1.44×10^-5^ | *ATM, MARK1, CHEK2, PRKAA2, MARK4, STK11* |
| GO:0016301:kinase activity | 2.47×10^-5^ | *ATM, MARK1, CHEK2, PRKAA2, MARK4, STK11, STRADB* |
| GO:0016772:transferase activity, transferring phosphorus-containing groups | 6.69×10^-5^ | *ATM, MARK1, CHEK2, PRKAA2, MARK4, STK11, STRADB* |
| GO:0035174:histone serine kinase activity | 1.12×10^-4^ | *ATM, PRKAA2* |
| GO:0050321:tau-protein kinase activity | 2.05×10^-3^ | *MARK1, MARK4* |
| GO:0035173:histone kinase activity | 2.91×10^-3^ | *ATM, PRKAA2* |
| GO:0005546:phosphatidylinositol-4,5-bisphosphate binding | 1.72×10^-2^ | *PARD3, MARK1* |
| GO:0004003:ATP-dependent DNA helicase activity | 2.08×10^-2^ | *NBN, MRE11A* |
| *Biological Process* |  |  |
| GO:0007050:cell cycle arrest | 1.43×10^-8^ | *ATM, CHEK2, MDC1, NBN, PRKAA2, STK11, STRADB, MRE11A* |
| GO:0006302:double-strand break repair | 3.09×10^-8^ | *ATM, CHEK2, MDC1, NBN, MRE11A, TP53BP1* |
| GO:0045786:negative regulation of cell cycle | 5.57×10^-8^ | *ATM, CHEK2, MDC1, NBN, PRKAA2, STK11, STRADB, MRE11A* |
| GO:0000724:double-strand break repair via homologous recombination | 1.17×10^-7^ | *ATM, MDC1, NBN, MRE11A, TP53BP1* |
| GO:0000725:recombinational repair | 1.28×10^-7^ | *ATM, MDC1, NBN, MRE11A, TP53BP1* |
| GO:0051726:regulation of cell cycle | 2.94×10^-6^ | *ATM, CHEK2, MDC1, NBN, PRKAA2, STK11, STRADB, MRE11A* |
| GO:0033554:cellular response to stress | 3.72×10^-6^ | *ATM, CHEK2, MDC1, NBN, PRKAA2, STK11, STRADB, MRE11A, TP53BP1* |
| GO:0006468:protein phosphorylation | 6.40×10^-6^ | *ATM, MARK1, CHEK2, NBN, PRKAA2, MARK4, STK11, STRADB, MRE11A* |
| GO:0007049:cell cycle | 1.33×10^-5^ | *PARD3, ATM, CHEK2, MDC1, NBN, PRKAA2, STK11, STRADB, MRE11A* |
| GO:0006974:response to DNA damage stimulus | 2.27×10^-5^ | *ATM, CHEK2, MDC1, NBN, STK11, MRE11A, TP53BP1* |
