# Supplementary material for: VarWalker: Personalized Mutation Network Analysis of Putative Cancer Genes from Next-Generation Sequencing Data
Source: PLoS Comput Biol. 2014 Feb 6;10(2):e1003460. doi: 10.1371/journal.pcbi.1003460 (PMC3916227; doi:10.1371/journal.pcbi.1003460)
Supplement: Table S7 — Significant interactions in which both interactors are encoded by genes from known LUAD genes, Cancer Gene Census (CGC), or kinase and involve one highly mutated gene and one rarely mutated gene (in bold), as determined by the threshold for mutation frequency, i.e., 182×5% = 9.1 samples. (DOCX) [file pcbi.1003460.s018.docx]

**Table S7**. Significant interactions in which both interactors are encoded by genes from known LUAD genes, Cancer Gene Census (CGC), or kinase, and involve one highly mutated gene and one rarely mutated gene (in bold), as determined by the threshold for mutation frequency, i.e., 182 × 5% = 9.1 samples.

| **Interactor A** | **# samples** | **Annotation for A** | **Interactor B** | **# samples** | **Annotation for B** |
| --- | --- | --- | --- | --- | --- |
| **JUN** | 1 | CGC | CREBBP | 11 | CGC |
| **FYN** | 1 | kinase | KDR | 11 | known LUAD genes, CGC, kinase |
| **IRAK1** | 1 | kinase | NTRK3 | 11 | known LUAD genes, CGC |
| **JUN** | 1 | CGC | NTRK3 | 11 | known LUAD genes, CGC |
| **HRAS** | 1 | known LUAD genes, CGC | BRAF | 13 | known LUAD genes, CGC, kinase |
| **IRAK1** | 1 | kinase | TLR4 | 13 | known LUAD genes |
| **AKT1** | 1 | known LUAD genes, CGC | ZNF521 | 14 | CGC |
| **MLLT1** | 1 | CGC | ARID1A | 15 | known LUAD genes, CGC |
| **SS18** | 1 | CGC | ARID1A | 15 | known LUAD genes, CGC |
| **SS18** | 1 | CGC | SMARCA4 | 16 | known LUAD genes, CGC |
| **HRAS** | 1 | known LUAD genes, CGC | NF1 | 19 | known LUAD genes, CGC |
| **MARK4** | 1 | kinase | STK11 | 27 | known LUAD genes, CGC |
| **BCL2** | 1 | CGC | KRAS | 49 | known LUAD genes, CGC |
| **BRCA1** | 1 | CGC | TP53 | 85 | known LUAD genes, CGC |
| **MDM2** | 1 | known LUAD genes, CGC | TP53 | 85 | known LUAD genes, CGC |
| **RAF1** | 2 | CGC, kinase | BRAF | 13 | known LUAD genes, CGC, kinase |
| **IRAK2** | 2 | kinase | TLR4 | 13 | known LUAD genes |
| **PDGFRB** | 2 | CGC | EGFR | 29 | known LUAD genes, CGC, kinase |
| **RAF1** | 2 | CGC, kinase | KRAS | 49 | known LUAD genes, CGC |
| **PAX5** | 3 | CGC | ZNF521 | 14 | CGC |
| **MLLT10** | 3 | CGC | ARID1A | 15 | known LUAD genes, CGC |
| **CHEK2** | 3 | CGC | ATM | 18 | known LUAD genes, CGC, kinase |
| **NUP98** | 4 | CGC | TPR | 10 | CGC |
| **NFE2L2** | 4 | CGC | KEAP1 | 22 | known LUAD genes |
| **ERBB2** | 4 | known LUAD genes, CGC | EGFR | 29 | known LUAD genes, CGC, kinase |
| **RALGDS** | 4 | CGC | KRAS | 49 | known LUAD genes, CGC |
| **MARK1** | 5 | kinase | STK11 | 27 | known LUAD genes, CGC |
| **CBL** | 5 | known LUAD genes, CGC | EGFR | 29 | known LUAD genes, CGC, kinase |
| **SMAD4** | 6 | known LUAD genes | CREBBP | 11 | CGC |
| **FLT1** | 6 | kinase | KDR | 11 | known LUAD genes, CGC, kinase |
| **SMAD4** | 6 | known LUAD genes | ZNF521 | 14 | CGC |
| **PBRM1** | 6 | CGC | SMARCA4 | 16 | known LUAD genes, CGC |
| **FLT4** | 7 | kinase | KDR | 11 | known LUAD genes, CGC, kinase |
| **EBF1** | 7 | CGC | ZNF521 | 14 | CGC |
