# Supplementary material for: VarWalker: Personalized Mutation Network Analysis of Putative Cancer Genes from Next-Generation Sequencing Data
Source: PLoS Comput Biol. 2014 Feb 6;10(2):e1003460. doi: 10.1371/journal.pcbi.1003460 (PMC3916227; doi:10.1371/journal.pcbi.1003460)
Supplement: Table S8 — Functional analysis of the melanoma mutation network: Top significant KEGG pathways ( p Bonferroni<10−6). (DOCX) [file pcbi.1003460.s019.docx]

**Table S8**. Functional analysis of the melanoma mutation network: top significant KEGG pathways (*p*_Bonferroni_ <10^-6^).

| **KEGG pathway** | **Count of genes** | ***p* value** | ***p*_Bonferroni_** |
| --- | --- | --- | --- |
| hsa05200:Pathways in cancer | 47 | 1.05×10^-17^ | 1.11×10^-15^ |
| hsa05214:Glioma | 22 | 3.30×10^-16^ | 3.53×10^-14^ |
| hsa05220:Chronic myeloid leukemia | 23 | 1.45×10^-15^ | 1.53×10^-13^ |
| hsa05219:Bladder cancer | 18 | 6.03×10^-15^ | 6.35×10^-13^ |
| hsa05215:Prostate cancer | 24 | 6.70×10^-15^ | 7.06×10^-13^ |
| hsa04510:Focal adhesion | 32 | 4.36×10^-13^ | 4.62×10^-11^ |
| hsa05211:Renal cell carcinoma | 20 | 6.67×10^-13^ | 7.07×10^-11^ |
| hsa05218:Melanoma | 20 | 8.85×10^-13^ | 9.38×10^-11^ |
| hsa04660:T cell receptor signaling pathway | 23 | 5.16×10^-12^ | 5.47×10^-10^ |
| hsa05213:Endometrial cancer | 17 | 5.75×10^-12^ | 6.10×10^-10^ |
| hsa05223:Non-small cell lung cancer | 17 | 1.10×10^-11^ | 1.17×10^-9^ |
| hsa05212:Pancreatic cancer | 19 | 1.30×10^-11^ | 1.37×10^-9^ |
| hsa04662:B cell receptor signaling pathway | 19 | 2.74×10^-11^ | 2.90×10^-9^ |
| hsa04722:Neurotrophin signaling pathway | 23 | 9.26×10^-11^ | 9.81×10^-9^ |
| hsa05210:Colorectal cancer | 19 | 2.09×10^-10^ | 2.21×10^-8^ |
| hsa04810:Regulation of actin cytoskeleton | 29 | 4.27×10^-10^ | 4.53×10^-8^ |
| hsa04664:Fc epsilon RI signaling pathway | 18 | 5.16×10^-10^ | 5.47×10^-8^ |
| hsa04012:ErbB signaling pathway | 18 | 3.11×10^-9^ | 3.30×10^-7^ |
| hsa05221:Acute myeloid leukemia | 15 | 4.53×10^-9^ | 4.80×10^-7^ |
