# Supplementary material for: VarWalker: Personalized Mutation Network Analysis of Putative Cancer Genes from Next-Generation Sequencing Data
Source: PLoS Comput Biol. 2014 Feb 6;10(2):e1003460. doi: 10.1371/journal.pcbi.1003460 (PMC3916227; doi:10.1371/journal.pcbi.1003460)
Supplement: Table S9 — Functional analysis of the melanoma mutation network: Top significant GO terms ( p Bonferroni<10−6). (DOCX) [file pcbi.1003460.s020.docx]

**Table S9**. Functional analysis of the melanoma mutation network: top significant GO terms (*p*_Bonferroni_ <10^-6^).

| **GO ID (BP)** | **GO term** | **Count** | ***p*-value** | ***p*_Bonferroni_** |
| --- | --- | --- | --- | --- |
| GO:0007167 | Enzyme linked receptor protein signaling pathway | 38 | 3.49×10^-15^ | 9.01×10^-12^ |
| GO:0070271 | Protein complex biogenesis | 45 | 2.05×10^-14^ | 5.38×10^-11^ |
| GO:0006461 | Protein complex assembly | 45 | 2.05×10^-14^ | 5.38×10^-11^ |
| GO:0010033 | Response to organic substance | 54 | 3.83×10^-14^ | 1.00×10^-10^ |
| GO:0007242 | Intracellular signaling cascade | 74 | 5.88×10^-14^ | 1.54×10^-10^ |
| GO:0042127 | Regulation of cell proliferation | 56 | 9.64×10^-14^ | 2.52×10^-10^ |
| GO:0051174 | Regulation of phosphorus metabolic process | 43 | 1.05×10^-13^ | 2.75×10^-10^ |
| GO:0019220 | Regulation of phosphate metabolic process | 43 | 1.05×10^-13^ | 2.75×10^-10^ |
| GO:0042325 | Regulation of phosphorylation | 42 | 1.26×10^-13^ | 3.30×10^-10^ |
| GO:0007169 | Transmembrane receptor protein tyrosine kinase signaling pathway | 29 | 2.72×10^-13^ | 7.13×10^-10^ |
| GO:0043549 | Regulation of kinase activity | 36 | 3.96×10^-13^ | 1.04×10^-9^ |
| GO:0009719 | Response to endogenous stimulus | 38 | 7.03×10^-13^ | 1.84×10^-9^ |
| GO:0051338 | Regulation of transferase activity | 36 | 1.32×10^-12^ | 3.45×10^-9^ |
| GO:0006468 | Protein amino acid phosphorylation | 49 | 1.57×10^-12^ | 4.12×10^-9^ |
| GO:0010604 | Positive regulation of macromolecule metabolic process | 55 | 1.02×10^-11^ | 2.66×10^-8^ |
| GO:0044093 | Positive regulation of molecular function | 44 | 1.40×10^-11^ | 3.68×10^-8^ |
| GO:0006796 | Phosphate metabolic process | 59 | 1.45×10^-11^ | 3.80×10^-8^ |
| GO:0006793 | Phosphorus metabolic process | 59 | 1.45×10^-11^ | 3.80×10^-8^ |
| GO:0043933 | Macromolecular complex subunit organization | 49 | 1.48×10^-11^ | 3.88×10^-8^ |
| GO:0009725 | Response to hormone stimulus | 34 | 2.07×10^-11^ | 5.42×10^-8^ |
| GO:0007610 | Behavior | 38 | 5.48×10^-11^ | 1.44×10^-7^ |
| GO:0007268 | Synaptic transmission | 30 | 5.52×10^-11^ | 1.45×10^-7^ |
| GO:0065003 | Macromolecular complex assembly | 46 | 6.78×10^-11^ | 1.78×10^-7^ |
| GO:0016310 | Phosphorylation | 51 | 8.94×10^-11^ | 2.34×10^-7^ |
| GO:0042981 | Regulation of apoptosis | 51 | 1.07×10^-10^ | 2.80×10^-7^ |
| GO:0007267 | Cell-cell signaling | 43 | 1.11×10^-10^ | 2.91×10^-7^ |
| GO:0060341 | Regulation of cellular localization | 27 | 1.11×10^-10^ | 2.91×10^-7^ |
| GO:0010557 | Positive regulation of macromolecule biosynthetic process | 45 | 1.37×10^-10^ | 3.60×10^-7^ |
| GO:0043067 | Regulation of programmed cell death | 51 | 1.52×10^-10^ | 3.98×10^-7^ |
| GO:0010941 | Regulation of cell death | 51 | 1.73×10^-10^ | 4.52×10^-7^ |
| GO:0031328 | Positive regulation of cellular biosynthetic process | 46 | 1.80×10^-10^ | 4.71×10^-7^ |
| GO:0016477 | Cell migration | 28 | 2.34×10^-10^ | 6.11×10^-7^ |
| GO:0009891 | Positive regulation of biosynthetic process | 46 | 2.89×10^-10^ | 7.56×10^-7^ |
| GO:0019899 | Enzyme binding | 39 | 3.58×10^-10^ | 2.06×10^-7^ |
