# Supplementary material for: VarWalker: Personalized Mutation Network Analysis of Putative Cancer Genes from Next-Generation Sequencing Data
Source: PLoS Comput Biol. 2014 Feb 6;10(2):e1003460. doi: 10.1371/journal.pcbi.1003460 (PMC3916227; doi:10.1371/journal.pcbi.1003460)
Supplement: Table S10 — Functional analysis of selected subgraphs in the melanoma mutation network: Top 10 significant pathways. (DOCX) [file pcbi.1003460.s021.docx]

**Table S10**. Functional analysis of selected subgraphs in the melanoma mutation network: top 10 significant pathways.

| **Pathway** | **Source** | ***p*_Bonferroni_** | **Genes in the first subgraph** |
| --- | --- | --- | --- |
| *The first subgraph* | |  |  |
| Genes involved in Signaling to p38 via RIT and RIN | MSigDB: C2.cp - Reactome | 3.38×10^-9^ | *YWHAB, NRAS, BRAF, MAP2K2, HRAS, RAF1* |
| Genes involved in Frs2-mediated activation | MSigDB: C2.cp - Reactome | 8.96×10^-9^ | *YWHAB, NRAS, BRAF, MAP2K2, HRAS, RAF1* |
| EGF receptor signaling pathway | PantherDB | 2.46×10^-7^ | *YWHAB, PIK3CA, NRAS, BRAF, MAP2K2, HRAS, NF1, RAF1, MRAS* |
| Genes involved in SHC-mediated signaling | MSigDB: C2.cp - Reactome | 2.59×10^-7^ | *YWHAB, NRAS, MAP2K2, HRAS, RAF1* |
| Genes involved in SOS-mediated signaling | MSigDB: C2.cp - Reactome | 4.20×10^-7^ | *YWHAB, NRAS, MAP2K2, HRAS, RAF1* |
| Genes involved in Grb2 events in EGFR signaling | MSigDB: C2.cp - Reactome | 4.20×10^-7^ | *YWHAB, NRAS, MAP2K2, HRAS, RAF1* |
| Genes involved in SHC-related events | MSigDB: C2.cp - Reactome | 6.51×10^-7^ | *YWHAB, NRAS, MAP2K2, HRAS, RAF1* |
| TGF-beta signaling pathway | PantherDB | 1.07×10^-6^ | *NRAS, HRAS, SMAD1, SMURF2, SMAD2, SMAD4, SMAD9, ACVR1* |
| Genes involved in Signaling to ERKs | MSigDB: C2.cp - Reactome | 1.43×10^-6^ | *YWHAB, NRAS, BRAF, MAP2K2, HRAS, RAF1* |
| Genes involved in Down-stream signal transduction | MSigDB: C2.cp - Reactome | 1.72×10^-6^ | *YWHAB, PIK3CA, NRAS, MAP2K2, HRAS, RAF1* |
| *The second subgraph* | |  |  |
| Signaling events mediated by VEGFR1 and VEGFR2 | NCI-Nature Curated | 6.92×10^-17^ | *CTNNB1, FLT1, FLT4, MAPK3, MAPK1, PLCG1, RAC1, KDR, PIK3R1, PRKACA, PTPN11, PTPN6, CDC42, SHC1, GRB2, VEGFC, GAB1* |
| B cell receptor signaling pathway | WikiPathways | 2.57×10^-15^ | *VAV1, MAPK4, MAPK1, PLCG1, RAC1, ATF2, CARD11, MAPK14, PIK3R1, PTPN11, CBL, PTPN6, SYK, GAB2, CDC42, SHC1, GRB2, GAB1* |
| Angiopoietin receptor Tie2-mediated signaling | NCI-Nature Curated | 2.01×10^-12^ | *ANGPT2, ANGPT1, ANGPT4, ITGA5, MAPK3, MAPK1, RAC1, MAPK14, PIK3R1, PTPN11, TEK, SHC1, GRB2* |
| scatter factor/hepatocyte growth factor signaling | Pathway Ontology | 1.87×10^-11^ | *MET, PLCG1, PIK3R1, PTPN11, CBL, SHC1, GRB2, GAB1* |
| TCR Signaling Pathway | WikiPathways | 2.60×10^-11^ | *VAV1, VAV3, MAPK3, MAPK1, PLCG1, ATF2, CARD11, MAPK14, PIK3R1, PTPN11, CBL, GAB2, CDC42, SHC1, GRB2* |
| Angiogenesis | PantherDB | 3.13×10^-11^ | *CTNNB1, ANGPT2, ANGPT1, MAPK6, MAPK3, MAPK1, PLCG1, AXIN2, AXIN1, KDR, MAPK14, PIK3R1, PTPN11, APC, PTPN6, TEK, SHC1, GRB2* |
| Signaling events mediated by Hepatocyte Growth Factor Receptor (c-Met) | NCI-Nature Curated | 4.78×10^-11^ | *CTNNB1, MET, MAPK3, MAPK1, PLCG1, RAC1, PIK3R1, PTPN11, APC, CBL, GAB2, CDC42, GRB2, GAB1* |
| Genes involved in Adherens junctions interactions | MSigDB: C2.cp - Reactome | 1.97×10^-10^ | *CTNNB1, CDH18, CDH10, CDH12, CDH15, CDH2, CDH7, CDH6, CDH9, JUP* |
| IL-4 signaling pathway | WikiPathways | 2.61×10^-10^ | *EP300, MAPK3, MAPK1, ATF2, MAPK14, PIK3R1, PTPN11, CBL, PTPN6, GAB2, SHC1, GRB2* |
| VEGFR3 signaling in lymphatic endothelium | NCI-Nature Curated | 8.45×10^-10^ | *FLT4, ITGA5, MAPK3, MAPK1, MAPK14, PIK3R1, GRB2, VEGFC, FIGF* |
