# Supplementary material for: VarWalker: Personalized Mutation Network Analysis of Putative Cancer Genes from Next-Generation Sequencing Data
Source: PLoS Comput Biol. 2014 Feb 6;10(2):e1003460. doi: 10.1371/journal.pcbi.1003460 (PMC3916227; doi:10.1371/journal.pcbi.1003460)
Supplement: Table S11 — Functional analysis of 94 overlapping genes between the lung adenocarcinoma consensus mutation network and the melanoma consensus mutation network (top 5 in each category). (DOCX) [file pcbi.1003460.s022.docx]

**Table S11**. Functional analysis of 94 overlapping genes between the lung adenocarcinoma consensus mutation network and the melanoma consensus mutation network (top 5 in each category).

| **GO ID/Pathway** | **Function** | **Count** | ***p*_Bonferroni_** |
| --- | --- | --- | --- |
| *GO: Molecular Function* | |  |  |
| GO:0019899 | Enzyme binding | 34 | 2.16×10^-13^ |
| GO:0005102 | Receptor binding | 34 | 3.03×10^-13^ |
| GO:0019902 | Phosphatase binding | 12 | 5.85×10^-9^ |
| GO:0019903 | Protein phosphatase binding | 10 | 6.89×10^-8^ |
| GO:0019900 | Kinase binding | 16 | 1.76×10^-6^ |
| *GO: Biological Process* | |  |  |
| GO:0007167 | Enzyme linked receptor protein signaling pathway | 33 | 2.21×10^-15^ |
| GO:0042127 | Regulation of cell proliferation | 37 | 1.04×10^-14^ |
| GO:0006468 | Protein phosphorylation | 37 | 1.15×10^-14^ |
| GO:0045935 | Positive regulation of nucleobase-containing compound metabolic process | 36 | 7.62×10^-14^ |
| GO:0009891 | Positive regulation of biosynthetic process | 37 | 1.67×10^-13^ |
| *Pathway* |  |  |  |
| BIOCARTA: RACCYCD pathway | Influence of Ras and Rho proteins on G1 to S transition | 9 | 1.26×10^-9^ |
| WP1984 | Integrated breast cancer pathway | 15 | 1.23×10^-8^ |
| vegfr1_2_pathway | Signaling events mediated by VEGFR1 and VEGFR2 | 11 | 1.74×10^-8^ |
| BIOCARTA: ARF pathway | Tumor suppressor Arf inhibits ribosomal biogenesis | 7 | 1.01×10^-7^ |
| WP179 | Cell cycle | 12 | 1.60×10^-7^ |
