# Supplementary material for: VarWalker: Personalized Mutation Network Analysis of Putative Cancer Genes from Next-Generation Sequencing Data
Source: PLoS Comput Biol. 2014 Feb 6;10(2):e1003460. doi: 10.1371/journal.pcbi.1003460 (PMC3916227; doi:10.1371/journal.pcbi.1003460)
Supplement: Text S1 — Detailed description of datasets and evaluation of the algorithm. (DOCX) [file pcbi.1003460.s023.docx]

**VarWalker: Personalized Mutation Network Analysis of Putative Cancer Genes from Next-generation Sequencing Data**

**Peilin Jia^1,4^, Zhongming Zhao^1,2,3,4,*^**

**1** Department of Biomedical Informatics, Vanderbilt University School of Medicine, Nashville, TN 37203, **2** Department of Cancer Biology, Vanderbilt University School of Medicine, Nashville, TN 37232, **3** Department of Psychiatry, Vanderbilt University School of Medicine, Nashville, TN 37232, **4** Center for Quantitative Sciences, Vanderbilt University Medical Center, Nashville, TN 37232

***Address correspondence and reprint requests to:**

Zhongming Zhao, Ph.D.

Department of Biomedical Informatics

Vanderbilt University School of Medicine

2525 West End Avenue, Suite 600

Nashville, TN 37203, USA

Phone: (615) 343-9158

FAX: (615) 936-8545

Email: [zhongming.zhao@vanderbilt.edu](mailto:zhongming.zhao@vanderbilt.edu)

**Supplementary Text S1**

Datasets

The lung adenocarcinoma (LUAD) dataset we used includes somatic mutations from 183 LUAD samples (95 males and 88 females) [1]. Among them, 27 were never smokers, 17 light smokers, 118 heavy smokers, and 21 had an unknown smoking history. A total of 90 patients were in stage I LUAD, 36 in stage II, 22 in stage III, 10 in stage IV, and 25 were unknown. More detailed information can be found in Imielinski et al., 2012 [1].

The melanoma somatic mutation dataset was obtained from 121 melanoma patients (67 males and 54 females) [2]. Briefly, these patients had an average age at diagnosis of 47 (range 16 to 85), including 15 primary tumors, 30 metastatic samples, and 76 short-term cultures derived from metastatic tumor tissue. Among them, 95 were cutaneous melanomas, 5 of acral, 2 mucosal, 1 uveal, and 18 were of unknown primary origin.

The validation datasets for both LUAD and melanoma were downloaded from The Cancer Genome Atlas (TCGA). For LUAD, a total of 518 samples were available (as of 7/18/2013) for WES data (Table S1). For the TCGA Skin Cutaneous Melanoma (SKCM), a total of 264 samples were available (as of 8/19/2013). We annotated these datasets in the same way as we did for the discovery datasets (see main text). Genes with non-silent deleterious mutations were defined as MutGenes and analyzed using VarWalker.

Personalized prioritization of mutation genes using the Random Walk with Restart algorithm

Step 1. Patient-specific assessment of MutGenes. To determine whether a sample-specific model or a universal model is appropriate for MutGenes assessment, we compared the retained MutGenes using each model. The sample-specific model was obtained using the sample’s MutGenes and thus, can successfully adjust sample-specific mutation profiles. However, the sample-specific model may be unreliable if a sample has only a few mutations. The universal model is obtained using all MutGenes in a cohort, but it may not account for the specificity of each single sample. In each sample, we obtained two sets of significant MutGenes assessed by the weighted resampling test, one using the sample-specific model and the other using the universal model. The differences were plotted and binned according to the number of MutGenes per sample. As shown in Supplementary Figure S11, samples with more MutGenes tend to have increased differences in the two sets of significant MutGenes, indicating that the sample-specific characteristics tend to play increased roles as the amount of MutGenes per sample increases. Accordingly, we selected 50 MutGenes as the cutoff for both LUAD and melanoma samples, as samples with greater than 50 MutGenes tend to clearly display differences between the remaining MutGenes when using a sample-specific model and when using the universal model.

Step 2. The sample-specific application of the Random Walk with Restart algorithm to search candidate interactors and MutGenes.

Step 3. Randomization-based evaluation of the candidate interactors.

Step 4. Construction of a consensus mutation subnetwork.

Performance evaluation

A systematic evaluation was performed for factors that may impact the results of VarWalker. First, the input genes per sample could include all MutGenes or recurrent MutGenes. Second, we explored three reference networks that may alter the results: the HPRD network [3], a network from Protein Interaction Network Analysis (PINA) platform [4], and a network based on functional pathway annotation [5]. PINA is a comprehensive PPI resource that integrates data from six public databases. The pathway-based network well represents biological correlations among genes but is only available for genes with pathway annotations. Third, the measurement of cDNA length could include the actual coding sequences. Alternatively, since only mutations that change the amino acid sequences will be of interest in the identification of somatic mutations, we counted the number of all possible non-silent mutations occurring in the cDNA regions and denoted it as an effective gene length. Fourth, a filtering step for genes that are two steps away from CGC genes may influence the resultant networks. We evaluated the performance of these factors in LUAD, which has a panel of known candidate genes. The recall rate was calculated as the number of captured known LUAD genes as a fraction of all known LUAD genes that are eligible for analysis. The proportion of captured known LUAD genes in the consensus network serves as another evaluation parameter. The overall results are presented in Figure S1. We employed two parameters to assess the performance: recall =$\frac{\text{\#captured known LUAD genes}}{\text{\#known LUAD genes}}$, and precision =$\frac{\text{\#captured known LUAD genes}}{\text{\#all genes in the consensus network}}$. After comparison, we found the overall performance achieved the best on the following conditions: (1) using all MutGenes as the input, (2) using the actual cDNA length as the measurement, (3) using the PPI network data from the Human Protein Reference Database (HPRD), and (4) filtering genes located more than two steps from CGC genes. All our follow-up analyses are based on this evaluation. In addition, we explored the number of MutGenes retained after the implementation of each step. This exploration revealed that mapping genes to the HPRD network removed the most genes (Figure S2).

References

1. Imielinski M, Berger AH, Hammerman PS, Hernandez B, Pugh TJ, et al. (2012) Mapping the hallmarks of lung adenocarcinoma with massively parallel sequencing. Cell 150: 1107-1120.

2. Hodis E, Watson IR, Kryukov GV, Arold ST, Imielinski M, et al. (2012) A landscape of driver mutations in melanoma. Cell 150: 251-263.

3. Keshava Prasad TS, Goel R, Kandasamy K, Keerthikumar S, Kumar S, et al. (2009) Human Protein Reference Database--2009 update. Nucleic Acids Res 37: D767-772.

4. Wu J, Vallenius T, Ovaska K, Westermarck J, Makela TP, et al. (2009) Integrated network analysis platform for protein-protein interactions. Nat Methods 6: 75-77.

5. Wu G, Feng X, Stein L (2010) A human functional protein interaction network and its application to cancer data analysis. Genome Biol 11: R53.

6. Huang da W, Sherman BT, Lempicki RA (2009) Bioinformatics enrichment tools: paths toward the comprehensive functional analysis of large gene lists. Nucleic Acids Res 37: 1-13.
